# A 90-Day Feeding Study in Rats to Assess the Safety of Genetically Engineered Pork

**DOI:** 10.1371/journal.pone.0165843

**Published:** 2016-11-03

**Authors:** Gao-jun Xiao, Sheng-Wang Jiang, Li-Li Qian, Chun-Bo Cai, Qing-qing Wang, De-Zun Ma, Biao Li, Shan-shan Xie, Wen-Tao Cui, Kui Li

**Affiliations:** 1 Institute of Animal Sciences, Chinese Academy of Agricultural Sciences, Beijing 100193, P. R. China; 2 State Key Laboratory of Agrobiotechnology, China Agricultural University, Beijing 100193, P. R. China; Max Delbruck Centrum fur Molekulare Medizin Berlin Buch, GERMANY

## Abstract

Our laboratory recently produced genetically engineered (GE) Meishan pigs containing a ZFN-edited *myostatin* loss-of-function mutant. These GE pigs develop and grow as normal as wild type pigs but produce pork with greater lean yield and lower fat mass. To assess any potential subchronic toxicity risks of this GE pork, a 90-day feeding study was conducted in Sprague-Dawley rats. Rats were randomly divided into five groups, and fed for 90 days with basic diet and basic diets formulated with low dose and high dose pork prepared from wild type pigs and GE pigs, respectively. Animal behaviors and clinical signs were monitored twice daily, and body weight and food consumption were measured and recorded weekly. At days 45 and 90, blood tests (lipid panel, electrolytes, parameters related to liver and kidney functions, and complete blood counts) were performed. Additionally, gross pathology and histopathological analyses were performed for major organs in each group. Data analysis shows that there were no significant differences in growth rate, food consumption, and blood test parameters between rat groups fed with GE pork and wild type pork. Although differences in some liver function parameters (such as aspartate aminotransferase, total proteins, albumin, and alkaline phosphatase) and white blood cell counts (such as lymphocyte percentage and monocyte percentage) were observed between rats fed with high dose GE pork and basic diet, all test results in rats fed with GE pork are in the normal range. Additionally, there are no apparent lesions noted in all organs isolated from rats in all five feeding groups on days 45 and 90. Overall, our results clearly indicate that food consumption of GE pork produced by ZFN-edited *myostatin* loss-of-function mutant pigs did not have any long-term adverse effects on the health status in rats.

## Introduction

Myostatin (*MSTN*), also known as growth and differentiation factor-8 (GDF-8), a member of the transforming growth factor-β (TGF-β) superfamily, is a dominant inhibitor of skeletal muscle development and growth. It has been well established that natural mutations in *MSTN* gene can lead to muscle hypertrophy or double-muscled (DM) phenotype in species such as cattle [1]. The DM cattle caused by natural mutations of *MSTN* loss-of-function have very strong skeletal muscle and contain much less fat. Experiments with *MSTN* knockout mice and the *in vivo* inhibition of *MSTN* activity by antagonists demonstrated that *MSTN* plays a negative regulatory role in muscle development and growth [2]. For example, *MSTN* knockout mice have a remarkable increase in muscle mass and significant decrease in fat compared to their corresponding wild-type littermates. Therefore, genetic manipulations of *MSTN* gene to generate loss-of-function *MSTN* mutations in livestock animals such as pigs have great potentials to improve meat quality for human consumption.

Gene editing is a new technology that specifically targets genome modifications and results in site specific DNA insertion, deletion or replacement in the genome of an organism [3]. Compared with traditional homologous recombination and embryonic stem cell technology-based gene targeting method, new gene editing technology has the advantage of site specific modification, high efficiency, low cost, and time saving, and it can be widely used in any species [4]. Although it is still in its infancy in developing new genetically engineered (GE) animals, gene editing technology is rapidly becoming an important molecular tool for basic research and application in the fields of life sciences and biomedicines [5–7]. Currently, there are three major gene editing technologies including nuclease-mediated zinc finger nucleases (ZFNs) [8], transcriptional activator like effector nucleases (TALENs) [9], and RNA-guided CRISPR-Cas nuclease [10]. ZFN technology has been successfully used in generating stable genetic mutants in a variety of species including rat [11], mouse [12], zebrafish [13], Drosophila [14], Arabidopsis [15], maize [16], and tobacco [17].

In 2015, Institute of Animal Sciences (IAS), Chinese Academy of Agricultural Sciences (CAAS) successfully produced GE Meishan pigs containing a ZFN-edited *MSTN* loss-of-function mutation [18]. The *MSTN* loss-of-function mutant in our GE pigs is due to the targeted deletion of 15 base pairs at exon 2 site, resulting in a premature translation termination. Compared with wild type (WT) pigs, the ZFN-edited *MSTN* mutant pigs have the same apparent phenotype as the DM Belgian cattle containing naturally occurring loss-of-function *MSTN* mutations, and produce improved quality pork with greater lean yield and lower fat mass. *MSTN* mutant pigs generated in our lab are as healthy as normal WT pigs. They develop and grow normally where they are raised and fed with the same normal diets as WT pigs, and they also have the same normal fertility as WT pigs without any abnormal pregnancy and other reproduction problems. Thus, these GE (*MSTN* mutant) pigs have an obvious advantage and potential to produce improved quality pork for human consumption.

Although ZFN technology has many advantages as a gene editing tool, there are indeed some concerns on unintended effects caused by off-target, cleavage of non-target sequences, and unexpected double strand break [19,20]. Polymerase Chain Reaction (PCR) analysis of our GE (*MSTN* loss-of-function mutant) pigs indicated that no off-target was observed and no integration of any ZFN vector DNA sequences into the porcine genome was seen. Additionally, results of blood testing, physiological characterizations, and clinical observations in our lab have proved that our GE pigs are healthy, no abnormality was observed, and no other unintended effects were noted.

Food safety of GE crops has been extensively assessed during last several decades. Up to date, all evidence and scientific data indicate that food from GE crops is safe for human consumption. However, very limited studies have been conducted to assess the safety or risks related to human consumption of meat produced by GE livestock animals, particularly GE pork. The 90-day feeding study in rats has a unique advantage in the evaluation of GE food safety. Rats have been widely used for safety assessment of GE food, particularly for food from GE crops [21–23]. Rats also have been used as a model for safety evaluation of meat derived from GE animals. For example, Bai et al had conducted a 90-day feeding experiment using rats to study the safety of meat derived from GE sheep overexpressing Toll-liker receptor-4 (TLR4) [24], and Liu et al also assessed the safety of GE beef produced by human lactoferrin transgenic cattle [25].

In this study, our aim is to assess the safety of pork from GE (*MSTN* loss-of-function mutant) pigs in rats during a 90-day feeding period. Based on the recommendations from the regulatory guidelines of the European Food Safety Authority (EFSA) [26] and the Chinese standards GB15193.13–2003, five groups of rats were fed for 90 days with basic diet, or basic diet formulated with low dose and high dose of pork from WT or GE pigs, respectively. Our results in this study clearly demonstrate that food consumption of GE pork produced by *MSTN* loss-of-function mutant pigs did not have any long-term adverse effects on health status in rats. Further studies will be required prior to market commercialization and human consumption of our GE pork.

## Materials and Methods

### Ethics statement

All experimental protocols related to animal work described in this study were reviewed and approved by the Institutional Animal Care and Use Committees (IACUC) at Institute of Animal Sciences, Chinese Academy of Agricultural Sciences and the Institute of Laboratory Animal Sciences, Chinese Academy of Medical Sciences Beijing, China. All experimental animals received adequate housing, feed, access to water and bedding. Daily observations were made by lab technicians to ensure that appropriate standards of animal care are being met. Feeding and management of experimental animals were conducted per standard operation procedures at animal facility located in New Drug Safety Evaluation and Research Center, the Institute of Laboratory Animal Sciences, Chinese Academy of Medical Sciences, Beijing, China.

### WT and GE pork preparation and nutritional analysis

Three WT pigs and three GE pigs were slaughtered, and meat from the back and legs were combined and used to prepare WT and GE pork, respectively. Pork was freeze-dried and milled at Processing Center, Institute of Agricultural Products, the Chinese Academy of Agricultural Sciences. The lyophilized powder of pork accounts for about 30% the weight of fresh meat. Nutrients including crude protein [27], total fiber [28], fats [29], calcium [30], phosphorus [31], ash [32] and amino acids [33] (see S1 Table) were analyzed in accordance with the standard methods of China. Table 1 is a summary of nutritional analysis of pork prepared from WT pigs and GE pigs.

**Table 1:** Nutritional analysis of Meishan pork.

Sample	Nutrient content(%)					
	Moisture	CP	EE	CF	Ash	P
**GE pork**	6.35	63.84	21.69	1.33	4.12	0.53
**WT pork**	4.68	68.43	20.94	1.26	4.10	0.64

CP: crude protein; EE: ether extract; CF: crude fiber.

### Preparation of rodent diets, nutritional analysis, and feeding dose

Commercial basic diet, which is meat free, is used as a baseline feed. Diets containing low dose and high dose pork were formulated by adding different amount of pork powder. The following five diets were prepared in this study: (1): basic diet (BD); (2) negative control 1 (NC1): low-dose WT pork; (3) negative control (NC2): high dose WT pork; (4) low dose GE pork (GE1); (5) high dose GE pork (GE2). All diet formulations were irradiated with ^60^Co to make specific pathogen free feed and were packed at Beijing Keao Feed Co. LTD (Beijing, China). Calculation of pork added to different diet formulations was based on the Chinese Dietary Guidelines, GB14924.3–2010, and Table 1. Two different amounts of pork were added to basic diet to make a low dose pork and a high dose pork. According to the "Chinese Dietary Guidelines" [34], an adult should consume 50-75g meat per day. By assuming a maximum intake of 75g meat per day per person and the average adult body weight of 60kg, a rat weighing 200g would need 0.25g meat per day. Since the pork used in this study was a lyophilized powder, which is 30% of fresh pork in weight, then the daily consumption of human equivalent amount by a 200g rat will be 0.075g of pork powder. Based on the recommendations from the Chinese Standard "GB15193.13–2015” for the 90-day feeding study, the low dose of pork is set at an amount equivalent to three times the amount of pork consumed by adult humans, and thus 0.225g pork powder per day per rat was formulated in basic diet to feed rats. Since each rat consumes 25g of total food powder per day, thus the percentage of pork powder added in the low dose diet is 0.9% (0.225/25 = 0.9%) in weight. For the high dose pork, since 4% of total fat in rat food is recommended, to avoid any potential problem that may be caused by nutritional imbalance, the maximum amount of pork equivalent to fifty times the amount of pork consumed by adult humans was selected in our study [26]. Thus, 3.75g powdered pork per day per rat (or 15% powdered pork) was formulated into the high dose diet for NC2 and GE2 groups. The final total fat in the high dose pork is 5–6%, which is already greater than the 4% specification for rat food. Similar high meat doses had been used in previous studies [24,35]. The nutrient contents of different rat diets are summarized in Table 2.

**Table 2:** Nutritional analysis of different rodent diets.

Nutrient (%)	Standard	Basic diet	WT pork		GE pork	
			0.9% powder(3-fold)	15% powder(50-fold)	0.9% powder(3-fold)	15% powder(50-fold)
**CP**	18	18.33	18.68	18.68	18.72	18.67
**EE**	4	6.19	5.67	5.66	5.66	5.73
**CF**	5	3.00	2.84	2.50	2.84	2.33
**Ash**	8	2.75	2.96	2.47	2.96	2.32
**Ca**	1–1.8	1.11	1.21	1.18	1.21	1.18
**P**	0.6–1.2	0.86	0.88	0.81	0.88	0.84

CP: crude protein; EE: ether extract; CF: crude fiber.

### Rats and their care

All experimental rats were of Sprague–Dawley strain, weaned, and purchased from Vital River Laboratory Animal Technology Co., Ltd. (Beijing, China). 100 rats, 50 males and 50 females, with body weight of 80-100g and age of 5 weeks, were divided into five groups (10 males and 10 females per group) and maintained under the following conditions: two rats with the same sex from the same group lived in one PVC cage, and were provided sterile food and drinking water *ad libitum;* temperature:23±2°C, relative humidity: 40–70%, automatic light control (12h light, 12h dark), and ≥15/h air exchange rate. All animal experiments were approved by Institutional Animal Care and Use Committee of the Institute of Laboratory Animal Sciences, Chinese Academy of Medical Sciences, Beijing, China.

### Clinical observations, food intake, and weight gain

Clinical signs were observed twice daily. Clinical signs include the appearance and behavior of animals, for example, changes in hair, skin, eyes, and mucous membranes, respiratory, central nervous system, limbs, amount of secretions and excretions. Body weight of each rat was weighed and recorded weekly. Amount of food consumption was monitored and recorded weekly. The mean daily food intake was calculated from the food weight consumed in a given period of time by two rats in a cage. Food weight was measured at the beginning of the feeding time and then measured again at the time of the next feeding. The mean food intake was calculated by weight difference between the two feedings divided by time (days) and number of rats (2).

### Blood tests

On days 45 and 90, blood samples were collected from each rat under anesthesia following a 16 hour fasting period. Complete blood counts and blood biochemistry were analyzed. Blood samples were collected in tubes containing EDTA-K2 for analysis of complete blood counts (CBC), and blood samples collected for analysis of blood biochemistry were centrifuged at 4500 rpm and room temperature for 10 min and the supernatants were saved for testing.

CBC was analyzed using Pentra DX 120 (ABX, France).

Analysis of CBC includes white blood cell (WBC), red blood cell (RBC), hemoglobin concentration (HGB), hematocrit (HCT), mean corpuscular volume (MCV), mean corpuscular hemoglobin(MCH), mean corpuscular hemoglobin concentration (MCHC), blood platelet count (PLT), mean platelet volume (MPV), red cell distribution width (RDW), lymphocyte percentage (LYM%), monocyte percentage (MON%).

Testing of blood biochemical parameters was performed using CoaLAB 1000 (Germany), Hitachi 7000 (Japan), and 9180 Electrolyte Analyzer (Roche), and includes the following: alanine aminotransferase (ALT), aspartate aminotransferase (AST), alkaline phosphatase (ALP), blood urea nitrogen (BUN), creatinine (CREA), glucose (GLU), albumin (ALB), total protein (TP), cholesterol (CHO), triglyceride (TG), high-density lipoprotein (HDL-C), low-density lipoprotein (LDL-C), calcium (Ca), chloride (Cl), and sodium (Na).

### Organ coefficient (ratio of organ to body weight) and histopathology

On day 45, 4 male rats and 4 female rats from each group, and on day 90, 6 male rats and 6 female rats from each group, were euthanized and dissected per protocols approved by IACUC of the Institute of Laboratory Animal Sciences, Chinese Academy of Medical Sciences, respectively. A complete gross necropsy was performed by visual inspection to check for any abnormity or lesion of organs. The weight of each organ was measured and the organ coefficient (ratio of organ to body weight) of each organ was calculated based on body weight. Organs measured in this study include heart, liver, kidney, adrenal, spleen, lung, thymus, and testis/ovary. Tissue from the following organs was fixed in 10% paraformaldehyde for histopathology analysis: brain, heart, lung, liver, kidney, spleen, stomach, thymus, adrenal gland, prostate, testis, ovary, uterus and skeletal muscle.

### Statistical analysis

Statistical analysis was designed to determine if differences exist at days 45 and 90 between GE1 and NC1, GE2 and NC2, GE1 and BD, and GE2 and BD. Data (expressed as mean ± SEM) of body weights, food consumption, lipid panel, levels of electrolytes, parameters related to liver and kidney functions, and organ coefficient (ratio of organ to body weight) were analyzed using Student t-test method (SAS release 8.1, SAS Institute Inc., Cary, NC). Complete blood counts and organ coefficients were expressed in mean ± SD, and analyzed using Student t-test (SAS release 8.1, SAS Institute Inc., Cary, NC). Differences between groups were considered significant if p < 0.05.

## Results

### Clinical observations

The animal health and welfare standards specified in EFSA [36] were followed for the 90-day feeding study in rats. Each rat in this study was clinically examined twice daily by monitoring each rat’s health status including animal behavior and amount of secretion and feces. No abnormity in animal behavior and clinical signs was noticed for any single rat in all five groups during the study period of 90 days.

### Body weight change and food consumption

**Fig 1:**
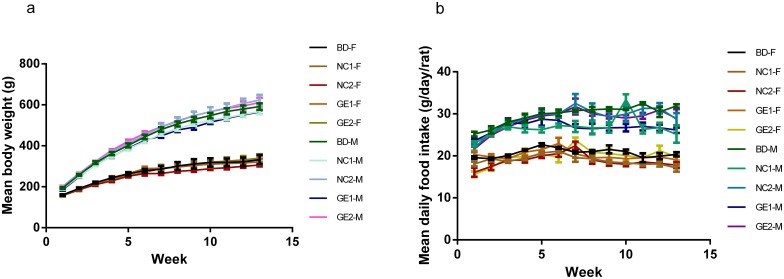
Body weight changes and food consumption during the 90-day feeding study in rats. (a) Changes in body weight; (b) food consumption. BD: basic diet; NC1: low-dose WT pork; NC2: high dose WT pork; GE1: low dose GE pork; GE2: high dose GE pork. All data are expressed in mean ± SD from six rats per sex per group at day 90.

Animal body weight and food consumption were measured and recorded weekly for each rat in all five groups during the 90-day study period. The pattern of growth curve (changes in body weight, see Fig 1a) and food consumption (Fig 1b) are very similar for all five groups, except the fact that male rats are heavier than female rats. Statistical analysis indicates that no significant differences were observed in body weight (Fig 1a) and food intake (Fig 1b) for male or female rats fed with basic diet, low and high dose pork from GE pigs or WT pigs.

### Lipid panel

**Fig 2:**
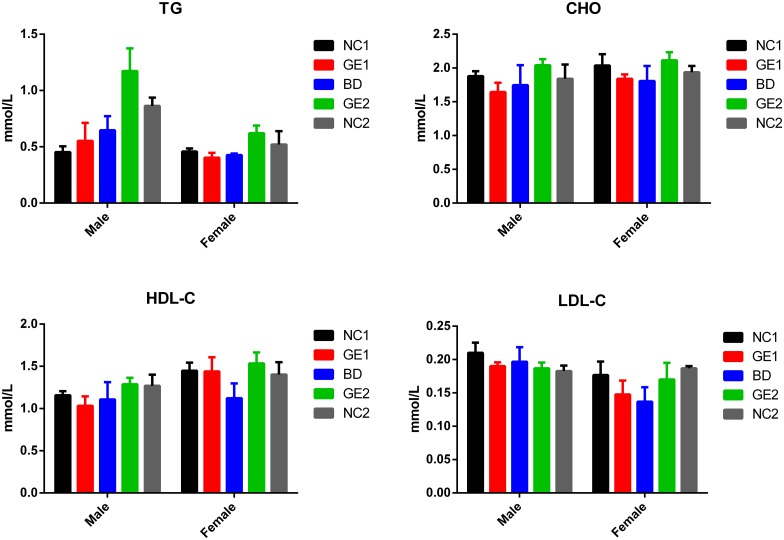
Test results of lipid panel. Blood samples were collected at day 90. BD: basic diet; NC1: low-dose WT pork; NC2: high dose WT pork; GE1: low dose GE pork; GE2: high dose GE pork. M: male; F: female. All data are expressed in mean ± SD from six rats per sex per group.

Lipids are important components of living cells and source of energy. Serum levels of total cholesterol (CHO), high density lipoprotein cholesterol (HDL-C), low density lipoprotein cholesterol (LDL-C), and triglycerides (TG) are widely used for cardiac risk assessment. The effect of feeding different diets on lipid panel was monitored by measuring and comparing serum levels of CHO, HDL-C, LDL-C, and TG. Results in Fig 2 show that there are no significant differences in serum levels of lipid panel among all five feeding groups at 90 days. For example, no differences were observed between rats fed with GE pork and rats fed with WT pork at both low dose and high dose. Furthermore, there are no differences between rats fed with pork (low and high doses) and rats fed with basic diet. The similar results were obtained at day 45 (see S2 Table). These data clearly indicate that all rats are healthy and there is no difference in terms of cardiovascular disease risks among rats fed with WT pork or GE (*MSTN* loss-of-function mutant) pork or basic diet.

### Electrolyte panel

**Fig 3:**
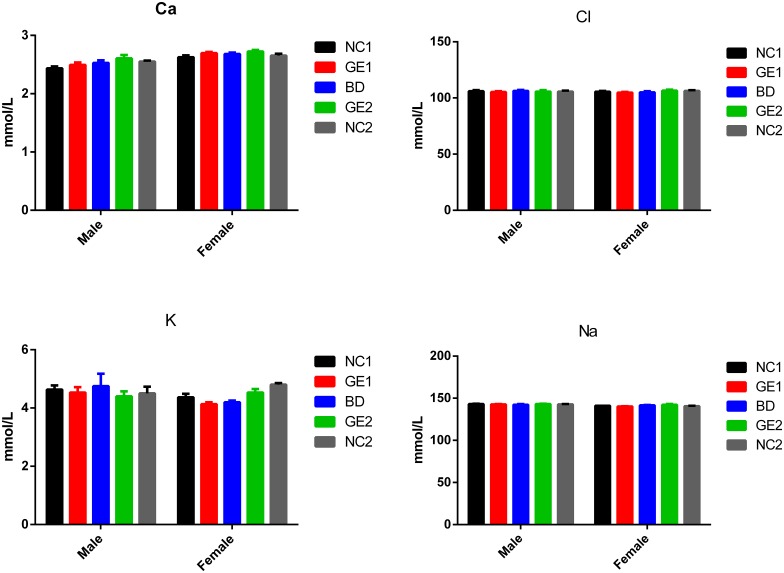
Results of serum electrolytes. Blood sample were collected at day 90. BD: basic diet; NC1: low-dose WT pork; NC2: high dose WT pork; GE1: low dose GE pork; GE2: high dose GE pork. M: male; F: female. All data are expressed in mean ± SD from six rats per sex per group.

Electrolytes are minerals that keep the body's fluids in balance and play many vital roles in normal body functions including heart rhythm, muscle contraction, blood pressure, osmotic pressure, pH maintenance, and nerve activity. Therefore, measurement of blood electrolytes such as sodium (Na^+^), potassium (K^+^), calcium (Ca^2+^), and chloride (Cl^-^) can provide useful healthy information. Fig 3 shows that there are no significant differences in serum blood levels of Na^+^, K^+^, Ca^2+^, and Cl^-^ among all five feeding groups at day 90. The similar results were obtained at day 45 (S3 Table). These results demonstrate that feeding GE pork or WT pork at either low dose or high dose has no effect on levels of electrolytes in blood.

### Liver function

**Fig 4:**
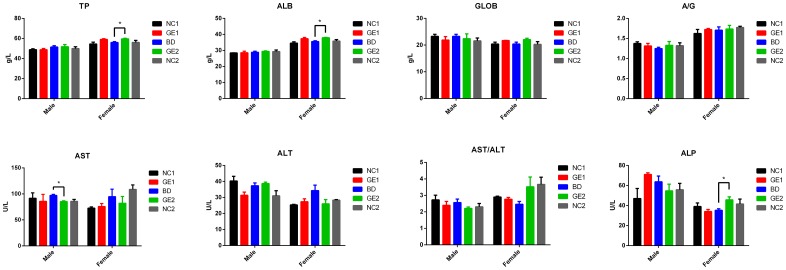
Test results of blood parameters related to liver function at day 90. Blood samples were collected at day 90. BD: basic diet; NC1: low-dose WT pork; NC2: high dose WT pork; GE1: low dose GE pork; GE2: high dose GE pork. M: male; F: female."*" Indicates a significant difference (p <0.05). All data are expressed in mean ± SD from six rats per sex per group.

Liver is one of the largest organs in the body. It is a metabolically active organ involved in many vital life functions including bile production, metabolism of fats, enzyme activation, synthesis of plasma proteins (such as albumin and clotting factors), and blood detoxification and purification. Therefore, monitoring liver function in the 90-day feeding study is a very important aspect of assessing the effect of GE pork on healthy status in rats. Blood tests for liver function include alanine aminotransferase (ALT), aspartate aminotransferase (AST), alkaline phosphatase (ALP), AST/ALT ratio, total protein (TP), globulins (GLOB), albumin (ALB), and A/G (ALB/GLOB) ratio. As shown in Fig 4, the test results of most parameters related to liver function are similar among all five feeding groups, particularly among rats fed with WT pork and GE pork day 90, although some differences were noted for a few parameters at day 90 between rats fed with basic diet and rats fed with GE pork. However, there are no significant differences in all test parameters between rats fed with basic diet and rats fed with GE pork or WT pork at day 45 (S4 Table). For example, at day 90, AST value (85.33 ± 2.05U/L) in male rats from GE2 group is significantly reduced compared with AST value (97.00 ± 2.94 U/L) (p <0.05) in male rats from BD group; levels of TP (59.59 ± 0.82g/L), ALB (37.88 ± 0.31 g/L), and ALP (45.67 ± 4.19 U/L) in female rats from GE2 group are significantly higher than corresponding levels (TP = 55.95 ± 1.28 g/L), ALB = 35.51 ± 0.71 g L, ALP = 35.33 ± 2.05 U/L) in female rats from BD group (p <0.05 for all three test results, see Fig 4). However, there are no significant differences between groups fed with WT pork and GE pork, and all reported values from GE2 groups fall in the normal range as observed for BD group rats.

**Fig 5:**
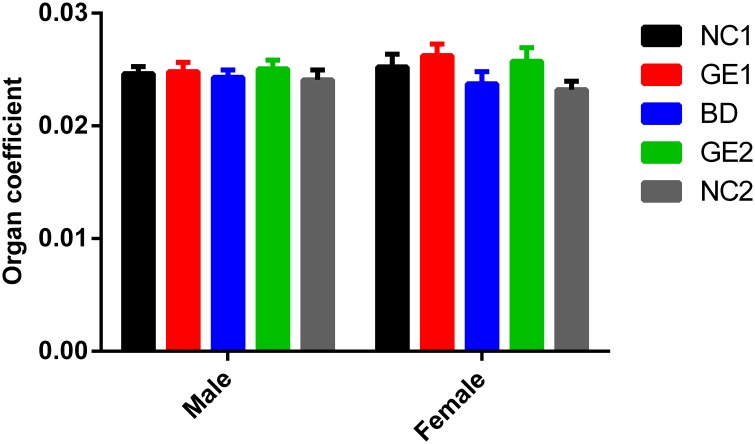
Relative liver weights (organ coefficients) at day 90. BD: basic diet; NC1: low-dose WT pork; NC2: high dose WT pork; GE1: low dose GE pork; GE2: high dose GE pork. M: male; F: female. All data are expressed in mean ± SD from six rats per sex per group.

Data analysis of liver weight and liver weight coefficient (liver weight /body weight ratio) from dissected rats (Fig 5) show that there is no difference observed in all five feeding groups at day 90. The similar results were obtained at day 45 (see S5 Table and S1 Fig).

**Fig 6:**
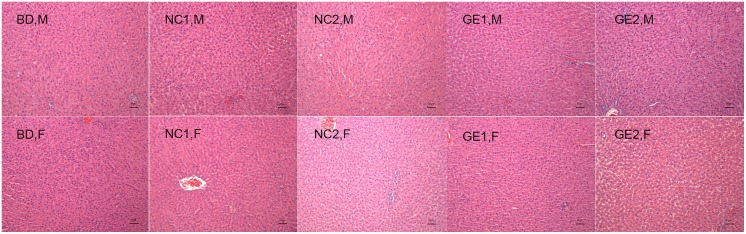
Liver histopathology at day 90. BD: basic diet; NC 1: low-dose WT pork; NC 2: high dose WT pork; GE 1: low dose GE pork; GE 2: high dose GE pork. M: male; F: female.

Necropsy analysis showed that no gross pathological and histopathologic findings related to WT pork and GE pork feeding were observed for the liver in all groups (Fig 6). A histopathological examination of liver tissue section indicates a clear lobular architecture, a normal proportion of cord and sinus, and no fibrous tissue proliferation and inflammatory cell infiltration observed in portal area.

All these data, including test results of parameters related to liver function, liver weight coefficient, and pathological and histopathologic observations, clearly indicate that feeding of WT or GE pork for 90 days has no effect on liver function.

### Kidney function

**Fig 7:**
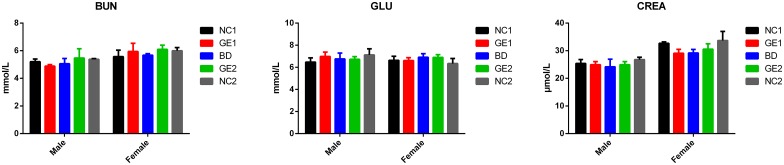
Test results of blood parameters (BUN, GLU, and CREA) related to kidney function at day 90. Blood samples were collected on day 90. BD: basic diet; NC1: low-dose WT pork; NC2: high dose WT pork; GE1: low dose GE pork; GE2: high dose GE pork. M: male; F: female. All data are expressed in mean ± SD from six rats per sex per group.

**Fig 8:**
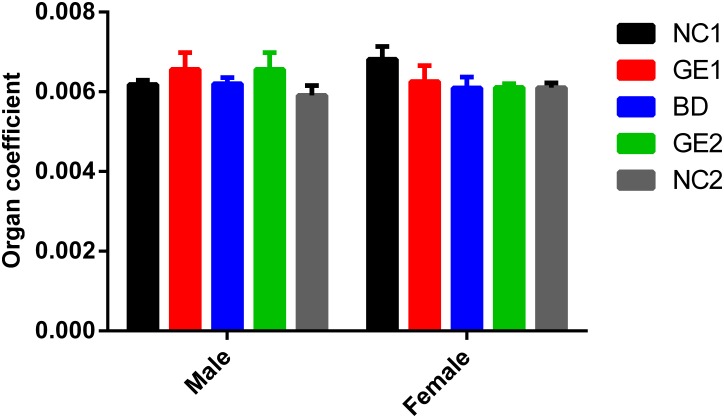
Kidney relative weight (organ coefficient) at day 90. BD: basic diet; NC1: low-dose WT pork; NC2: high dose WT pork; GE1: low dose GE pork; GE2: high dose GE pork. M: male; F: female. All data are expressed in mean ± SD from six rats per sex per group.

The kidney is an important organ that performs many essential functions including removing waste products from blood and regulating water fluid levels, producing hormones, regulating electrolytes and blood pressure. Any damage to the structure and function of kidneys will have an impact on these functions, thereby subsequently affecting the health of the body. Widely used tests for renal function include blood urea nitrogen (BUN), creatinine clearance (CREA), and glucose (GLU). Results shown in Fig 7 demonstrate that renal function test parameters are similar among all five feeding groups Additionally, there is no difference observed for kidney coefficient (kidney weight/body weight ratio) from dissected rats (Fig 8) in all five groups. All data clearly indicate that feeding of WT and GE pork has no effect on kidney function.

**Fig 9:**
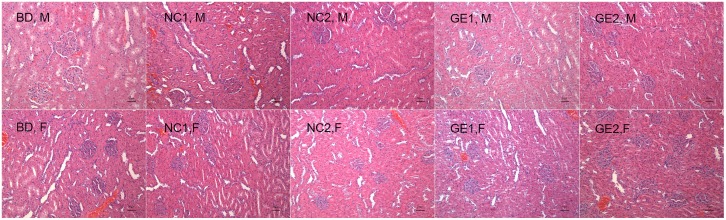
Kidney histopathology at day 90. BD: basic diet; NC1: low-dose WT pork; NC2: high dose WT pork; GE1: low dose GE pork; GE2: high dose GE pork. M: male; F: female.

No gross pathological and histopathologic findings related to WT pork and GE pork feeding were observed for the kidney in all groups during the necropsy (Fig 9). Pathological HE staining results shown in Fig 9 indicate that kidney cortex and medulla from GE1, GE2 groups are clear, without any abnormality being noted for glomeruli, tubules, collecting duct, mesenchyme and pelvis mucosa. The similar results were obtained at day 45 for serum parameters related to kidney function, kidney weight and kidney weight coefficient, and histopathology (see S6 and S7 Tables and S1 Fig). In summary, all results from both day 45 and day 90 clearly demonstrate that GE pork did not adversely affect kidney function in rats.

### Hematology

The complete blood count (CBC) is often used as a broad screening test to determine an individual's general health status and detect a wide range of disorders such as anemia, infection and leukemia. Abnormal increases or decreases in blood cell counts are a reflection of the healthy status. As seen in Tables 3 and 4, there are no significant differences in complete blood counts among all five feeding groups at days 45 and 90. Although some differences were noted in lymphocyte percentage (LYM%) and monocyte percentage (MON%). At day 90, the mean LYM% (an independently measured value to reflect peripheral lymphocyte count and a possible nutritional marker) in GE2 group male rats (55.17 ± 4.13) is significantly lower (p <0.05) than the corresponding value (65.47 ± 0.95) in the BD group male rats, but there was no significant difference in LYM% values between male rats from GE2 group and male rats from NC2 group. At day 45, the mean MON% (0.37 ± 0.05) in GE2 group female rats is significantly lower than the corresponding MON% value (0.53 ± 0.04) in BD group female rats (p <0.05), but had no difference when compared with NC2 group female rats.

**Table 3:** Summary of blood parameters of male rat.

	Test results at day 45					Test results at day 90				
	BD	NC1	NC2	GE1	GE2	BD	NC1	NC2	GE1	GE2
WBC	6.93±0.26	5.30±0.99	5.47±0.47	6.43±0.05	6.57±1.58	5.33±0.81	3.57±0.79	5.77±1.25	5.10±1.74	4.70±0.45
RBC	7.55±0.05	7.21±0.18	7.56±0.26	7.35±0.14	7.48±0.15	8.11±0.07	7.69±0.21	7.75±0.32	7.98±0.36	7.99±0.36
HGB	150.00±2.16	142.67±3.68	146.0±2.94	142.50±6.26	150.0±6.53	146.33±3.40	140.33±2.36	142.0±5.54	141.0±3.46	150.0±2.16
HCT	0.42±0.01	0.40±0.01	0.41±0.01	0.41±0.01	0.43±±0.01	0.42±0.01	0.41±0.002	0.42±0.02	0.41±0.01	0.43±0.005
MCV	56.33±0.47	57.0±0.71	56.75±1.09	57.0±1.0	57.75±1.09	53.0±0.82	53.25±0.43	53.67±1.25	53.67±0.94	54.0±2.16
MCH	19.93±0.12	20.13±0.42	20.18±0.57	20.23±0.48	20.47±0.25	18.50±0.37	18.27±0.24	18.13±0.61	18.53±0.37	18.80±0.67
MCHC	354.75±2.05	355.33±2.87	355.75±4.92	355.25±9.65	359.0±3.54	347.50±2.18	344.33±2.87	340.33±4.50	344.25±3.56	349.33±0.94
RDW	12.05±0.29	11.57±0.52	11.33±0.19	12.03±0.09	11.47±0.25	13.27±0.26	12.88±0.23	13.13±0.46	12.90±0.54	13.47±0.79
PLT	927.33±73.10	929.0±6.98	874.25±71.77	815.33±85.97	898.33±14.97	754.67±37.28	777.67±23.70	851.67±7.72	783.67±24.07	799.33±56.04
MPV	6.40±0.08	6.08±0.18	6.30±0.22	6.18±0.31	6.30±0.35	6.30±0.12	6.50±0.14	6.53±0.29	6.38±0.49	6.23±0.24
PCT	0.59±0.04	0.54±0.01	0.60±0.04	0.53±0.05	0.55±0.01	0.50±0.04	0.48±0.01	0.56±0.02	0.53±0.02	0.50±0.05
LYM%	73.78±4.35	79.58±0.57	77.13±2.57	74.40±3.75	78.45±3.48	65.47±0.95	61.03±2.72	62.20±1.57	59.30±7.57	**55.17±4.13**^a^
MON%	0.37±0.09	0.38±0.08	0.55±0.15	0.50±0.0	0.27±0.09	0.70±0.08	0.93±0.17	0.60±0.14	0.53±0.19	0.67±0.24
NEU%	23.30±3.81	18.3±0.73	21.73±3.54	22.60±3.31	19.70±2.34	31.27±0.66	34.43±3.49	33.87±2.57	34.67±11.52	37.80±3.77

BD: basic diet; NC1: low-dose WT-pork; NC2: high-dose WT-pork; GE1: low dose GE pork; GE2: high dose GE pork.

^a^ Significant differences compared with BD group (p<0.05). All data are expressed in mean ± SD from four rats per group at day 45 and six rats per group at day 90.

**Table 4:** Summary of blood parameters for female rats.

	Test results at day 45					Test results at day 90				
	BD	NC1	NC2	GE1	GE2	BD	NC1	NC2	GE1	GE2
WBC	2.83±0.78	3.63±0.17	4.90±1.13	3.37±0.76	4.47±0.56	4.27±0.50	3.70±0.43	4.90±0.62	3.0±0.60	4.80±0.85
RBC	6.84±0.32	6.67±0.13	6.59±0.27	6.56±0.20	6.38±0.26	6.85±0.09	7.00±0.10	7.19±0.19	6.93±0.16	7.00±0.39
HGB	140.0±3.27	134.33±2.05	137.75±5.40	131.50±6.73	139.33±7.72	133.0±2.94	133.75±2.34	134.33±6.60	134.0±2.92	133.0±1.41
HCT	0.38±0.01	0.36±0.01	0.38±0.01	0.36±0.02	0.36±0.01	0.38±0.01	0.38±0.01	0.39±0.004	0.38±0.001	0.38±0.006
MCV	55.75±1.48	55.33±2.05	56.67±1.70	55.67±0.94	56.75±0.83	55.25±1.30	55.0±0.71	54.25±1.64	54.75±1.48	55.75±1.48
MCH	20.63±0.51	20.03±0.80	20.28±0.68	20.03±0.48	20.53±0.24	19.53±0.52	19.10±0.48	19.20±0.65	18.80±0.22	19.13±0.47
MCHC	368.67±1.25	370.67±4.11	367.0±2.16	364.33±2.05	367.25±3.56	350.33±2.05	348.25±3.70	349.67±5.44	347.25±0.8	350.33±1.89
RDW	10.35±0.44	10.35±0.15	10.50±00.22	10.63±0.17	10.25±0.21	12.48±0.19	12.73±0.61	12.37±0.29	12.28±0.19	12.43±0.25
PLT	888.0±117.45	750.0±75.70	762.67±12.47	832.0±16.57	790.0±92.84	738.0±258.48	771.33±51.41	714.33±82.52	740.67±95.86	759.0±290.0
MPV	6.30±0.29	6.15±0.39	6.35±0.45	6.60±0.16	6.35±0.49	6.50±0.36	6.60±0.14	6.83±0.48	6.27±0.45	6.47±0.05
PCT	0.54±0.05	0.49±0.03	0.51±0.01	0.54±0.02	0.51±0.01	0.47±0.15	0.51±0.06	0.47±0.03	0.46±0.04	0.49±0.19
LYM%	75.77±3.53	74.53±1.69	70.33±7.80	69.93±7.95	81.93±1.92	68.25±12.72	69.40±6.38	68.80±1.40	68.37±3.97	63.53±9.37
MON%	0.53±0.04	0.35±0.11	0.53±0.09	0.50±0.08	**0.37±0.05**^a^	0.50±0.08	0.53±0.20	0.50±0.16	0.48±0.11	0.50±0.08
NEU%	19.57±2.47	23.23±1.51	23.73±5.98	24.07±6.49	15.90±1.74	25.20±6.20	26.23±4.50	30.10±6.36	27.73±3.99	32.57±8.75

BD: basic diet; NC1: low-dose WT pork; NC2: high dose WT pork; GE1: low dose GE pork; GE2: high dose GE pork.

^a^ Significant differences compared with BD group (p<0.05). All data are expressed in mean ± SD from four rats per group at day 45 and six rats per group at day 90.

### Organ relative weight (organ coefficient)

On day 45, 4 male rats and 4 female rats from each group, and on day 90, 6 male rats and 6 female rats from each group, were euthanized and dissected, respectively. Organs of each rat were weighed and the relative weight index (organ coefficient, which equals to organ weight/body weight) was calculated. As shown in Table 5, there was no significant difference in organ relative weight index (organ coefficients) among all five feeding groups for a variety of organs at days 45 and 90.

**Table 5:** Relative organ weights at day 45 and day 90.

	Results at day 45					Results at day 90				
	BD	NC1	NC2	GE1	GE2	BD	NC1	NC2	GE1	GE2
Male rats										
Heart	0.330±0.035	0.349±0.037	0.329±0.009	0.337±0.019	0.331±0.018	0.285±0.043	0.280±0.011	0.25±0.032	0.300±0.021	0.296±0.030
Spleen	0.161±0.019	0.177±0.016	0.178±0.024	0.187±0.027	0.190±0.025	0.180±0.031	0.150±0.018	0.161±0.010	0.155±0.011	0.172±0.005
Lungs	0.364±0.034	0.388±0.043	0.385±0.037	0.393±0.062	0.365±0.047	0.393±0.049	0.388±0.040	0.450±0.091	0.382±0.049	0.401±0.059
Adrenal	0.022±0.009	0.029±0.011	0.021±0.006	0.022±0.003	0.036±0.019	0.016±0.009	0.019±0.008	0.013±0.001	0.019±0.007	0.015±0.004
Thymus	0.147±0.041	0.152±0.027	0.161±0.023	0.201±0.055	0.157±0.009	0.069±0.014	0.105±0.046	0.079±0.024	0.081±0.019	0.086±0.012
Testis	0.767±0.080	0.814±0.080	0.736±0.033	0.807±0.100	0.759±0.045	0.614±0.034	0.635±0.083	0.572±0.074	0.645±0.041	0.623±0.041
Female rats										
Heart	0.350±0.066	0.335±0.030	0.317±0.022	0.327±0.027	0.332±0.018	0.289±0.014	0.318±0.043	0.292±0.025	0.316±0.014	0.327±0.047
Spleen	0.203±0.031	0.205±0.022	0.198±0.030	0.192±0.023	0.213±0.026	0.203±0.035	0.182±0.014	0.190±0.022	0.188±0.042	0.199±0.048
Lungs	0.491±0.071	0.551±0.111	0.471±0.048	0.457±0.052	0.557±0.109	0.510±0.091	0.539±0.088	0.538±0.082	0.513±0.082	0.510±0.067
Adrenal	0.043±0.029	0.039±0.012	0.048±0.014	0.036±0.010	0.057±0.006	0.031±0.011	0.027±0.010	0.047±0.024	0.023±0.005	0.029±0.008
Thymus	0.150±0.008	0.134±0.053	0.148±0.063	0.160±0.025	0.193±0.045	0.097±0.009	0.126±0.033	0.121±0.050	0.128±0.030	0.140±0.039
Ovary	0.079±0.020	0.070±0.013	0.092±0.029	0.083±0.034	0.091±0.025	0.088±0.019	0.092±0.013	0.098±0.023	0.086±0.029	0.078±0.030

BD: basic diet; NC1: low-dose WT pork; NC2: high dose WT pork; GE1: low dose GE pork; GE2: high dose GE pork. M: male; F: female. All data are expressed in mean ± SD from four rats per group at day 45 and six rats per group at day 90.

### Histopathology

Histopathological examination under the microscope of HE stained tissue section from all key organs showed that there were not any obvious lesions noted for the following organs from selected rats of all five feeding groups: brain, heart, lung, spleen, stomach, thymus, adrenal gland, prostate, testis, ovary, uterus and skeletal muscle (See S2–S13 Figs).

## Discussion

Myostatin is a very potent regulatory protein that is present in muscle, serum, and other tissues at very low level [2]. It inhibits muscle growth in a variety of species. Naturally occurring loss-of-function *MSTN* mutations have been reported and identified in cattle [1], dog [37], sheep [38] and humans [39]. However, naturally occurring loss-of-function *MSTN* mutations have not been reported in pigs. In recent years, gene-editing technologies have been widely used as an alternative to traditional breeding method. Of these gene editing technologies, ZFN is one the earliest and most widely used technology in animal and plant breeding.

Pork is a major meat diet in China, and from a health point of view, an improved high quality of pork with higher lean meat yield combined with lower body fat is highly desirable. Meishan pigs are a locally famous breed in China, and are well known for their high prolificacy and early sexual maturity, but the breed has a high percentage of carcass fat and poor feed efficiency. These unique qualities make Meishan pigs a suitable model to test if a mutation similar to naturally occurring *MSTN* loss-of-function and DM phenotype can be created by using gene-editing tool to improve the quality of pork. Along this line, our lab recently successfully generated *MSTN* loss-of-function mutant pigs by specifically targeting the exon 2 site of porcine *MSTN* gene using ZFN technology and somatic cell nucleus transfer. Our previous data have already demonstrated that such loss-of-function *MSTN* mutant GE pigs develop and grow normally; and there were not any off-targets or unintended effects being introduced by ZFN technology [18]. Compared to WT pork, *MSTN* loss-of-function GE pigs produce high quality pork with greater lean yield and lower fat mass.

It is evident that no foreign proteins were introduced in the *MSTN* loss-of-function pigs, thus, we would expect that the GE pork from such pigs will be as safe as WT pork. From a nutritional point of view, our GE pork will be healthier to human consumption due to the fact that it contains lower fat content. Even though our GE pork is safe based on our previous data from GE pigs, it is still very important to conduct safety studies in model animals such as rats to assess and confirm the safety of our GE pork. In this study, for the first time, we designed and conducted a 90-day feeding study in rats based on the recommendations from the guidelines of EFSA to assess the sub-chronic toxicity risks of GE pork produced by *MSTN* loss-of-function GE pigs. Data collected from body and organ weights, test results of liver and kidney functions, complete blood counts, and histopathology analysis, clearly indicate there are no differences in rats fed with WT pork and GE pork at both low and high doses.

From the test results of liver function parameters, it is noted that at day 90, AST values in male rats from GE2 group were significantly lower than AST values in male rats from BD group, but no significant difference was noted when compared with NC2 group. There is a decreasing trend in AST values in NC2 group rats compared with BD group rats. By analyzing combined data from GE1, GE2, NC1, and NC2 groups, we also noted that there is a decreasing trend in AST values in pork-fed groups. However, there is no significant difference in the AST/ALT ratio between pork-fed groups and basic diet group. Therefore, we speculate that feeding pork either from WT pigs or GE pigs could lead to a decrease in AST values. There is a significant increase in TP, ALB, and ALP in female rats from GE2 group compared with BD group, but no significant differences in these values between GE2 between NC2 groups. This observation is consistent with the results of a previous similar experiments [25]. Based on the normal ALP range (24–82 U/L) of rats fed with basic diet, it is likely that an increase in female serum ALP content may be due to these rats’ sensitivity to food ingredients. A/G ratio is a good indicator of liver function. Although TP and ALB in GE2 group were significantly higher than in BD group, there is no significant difference in A/G ratio between these groups, indicating that the change in TP and ALB does not adversely affect liver function.

Slight differences in LYM% were observed between rats fed with high dose GE pork and basic diet, but the LYM% values in all GE2 group rats still fall within the normal range of LYM% for rats. Although, at day 45, the mean MON% in GE2 group female rats is significantly lower than the corresponding MON% value in BD group female rats, no difference was noted when GE2 group was compared to NC2 group. Further, all MON% values in the GE2 group female rats are in the normal range for rats. The difference in MON% between GE2 and BD groups disappeared on day 90. Thus it is likely that the difference observed at day 45 between GE2 and BD groups could be an unexpected error. Visual inspection of rat immune organs did not find significant differences between GE2 and BD groups, so it is clear that feeding of GE pork did not adversely affect parameters of blood testing.

Additionally, there are no apparent lesions noted in all organs isolated from rats in all five feeding groups on day 45 and day 90. Overall, our results clearly indicate that that no adverse effects or subchronic toxic effects were identified in rats fed with GE pork in comparison with WT pork after the 90-day feeding period.

## Conclusion

In summary, our results on body weight and organ weight, test parameters related liver and kidney functions, white blood cell counts, and histopathology in this 90-day feeding study demonstrated that long-term food consumption of GE pork produced by *MSTN* loss-of-function mutant Meishan pigs did not have any adverse effects on health status in rats. These data also provided useful scientific evidence to support the future commercialization of GE pork produced by ZFN-edited *MSTN* loss-of-function mutant pigs for human consumption.

## Supporting Information

S1 Fig Liver and Kidney histopathology at day 45. BD: basic diet; NC1: low-dose WT pork; NC2: high dose WT pork; GE1: low dose GE pork; GE2: high dose GE pork. M: male; F: female.
(TIF)

S2 Fig Brain histopathology at day 45 and day 90. BD: basic diet; NC1: low-dose WT pork; NC2: high dose WT pork; GE1: low dose GE pork; GE2: high dose GE pork. M: male; F: female.
(TIF)

S3 Fig Heart histopathology at day 45 and day 90. BD: basic diet; NC1: low-dose WT pork; NC2: high dose WT pork; GE1: low dose GE pork; GE2: high dose GE pork. M: male; F: female.
(TIF)

S4 Fig Lung histopathology at day 45 and day 90. BD: basic diet; NC1: low-dose WT pork; NC2: high dose WT pork; GE1: low dose GE pork; GE2: high dose GE pork. M: male; F: female.
(TIF)

S5 Fig Spleen histopathology at day 45 and day 90. BD: basic diet; NC1: low-dose WT pork; NC2: high dose WT pork; GE1: low dose GE pork; GE2: high dose GE pork. M: male; F: female.
(TIF)

S6 Fig Stomach histopathology at day 45 and day 90. BD: basic diet; NC1: low-dose WT pork; NC2: high dose WT pork; GE1: low dose GE pork; GE2: high dose GE pork. M: male; F: female.
(TIF)

S7 Fig Thymus histopathology at day 45 and day 90. BD: basic diet; NC1: low-dose WT pork; NC2: high dose WT pork; GE1: low dose GE pork; GE2: high dose GE pork. M: male; F: female.
(TIF)

S8 Fig Adrenal gland histopathology at day 45 and day 90. BD: basic diet; NC1: low-dose WT pork; NC2: high dose WT pork; GE1: low dose GE pork; GE2: high dose GE pork. M: male; F: female.
(TIF)

S9 Fig Prostate histopathology at day 45 and day 90. BD: basic diet; NC1: low-dose WT pork; NC2: high dose WT pork; GE1: low dose GE pork; GE2: high dose GE pork. M: male; F: female.
(TIF)

S10 Fig Testis histopathology at day 45 and day 90. BD: basic diet; NC1: low-dose WT pork; NC2: high dose WT pork; GE1: low dose GE pork; GE2: high dose GE pork. M: male; F: female.
(TIF)

S11 Fig Ovary histopathology at day 45 and day 90. BD: basic diet; NC1: low-dose WT pork; NC2: high dose WT pork; GE1: low dose GE pork; GE2: high dose GE pork. M: male; F: female.
(TIF)

S12 Fig Uterus histopathology at day 45 and day 90. BD: basic diet; NC1: low-dose WT pork; NC2: high dose WT pork; GE1: low dose GE pork; GE2: high dose GE pork. M: male; F: female.
(TIF)

S13 Fig Skeletal muscle histopathology at day 45 and day 90. BD: basic diet; NC1: low-dose WT pork; NC2: high dose WT pork; GE1: low dose GE pork; GE2: high dose GE pork. M: male; F: female.
(TIF)

S1 Table Percentage of amino acids in pork produced by wild type and GE pigs. GE: genetically engineered; WT: wild type.
(DOCX)

S2 Table Test results (mean ± SD) of serum lipid panel at day 45. BD: basic diet; NC1: low-dose WT pork; NC2: high dose WT pork; GE1: low dose GE pork; GE2: high dose GE pork. All data are expressed in mean ± SD from four rats per sex per group.
(DOCX)

S3 Table Test results (mean ± SD) of serum electrolytes at day 45. BD: basic diet; NC1: low-dose WT pork; NC2: high dose WT pork; GE1: low dose GE pork; GE2: high dose GE pork. All data are expressed in mean ± SD from four rats per sex per group.
(DOCX)

S4 Table Test results (mean ± SD) of blood parameters related to liver function serum electrolytes at day 45. BD: basic diet; NC1: low-dose WT pork; NC2: high dose WT pork; GE1: low dose GE pork; GE2: high dose GE pork. All data are expressed in mean ± SD from four rats per sex per group.
(DOCX)

S5 Table Results (mean ± SD) of liver weight and liver weight coefficient (liver weight /body weight ratio) at days 45. BD: basic diet; NC1: low-dose WT pork; NC2: high dose WT pork; GE1: low dose GE pork; GE2: high dose GE pork. All data are expressed in mean ± SD from four rats per sex per group.
(DOCX)

S6 Table Test results (mean ± SD) of blood parameters related to renal function at days 45. BD: basic diet; NC1: low-dose WT pork; NC2: high dose WT pork; GE1: low dose GE pork; GE2: high dose GE pork. All data are expressed in mean ± SD from four rats per sex per group.
(DOCX)

S7 Table Results (mean ± SD) of kidney weight and kidney weight coefficient (liver weight /body weight ratio) at days 45. BD: basic diet; NC1: low-dose WT pork; NC2: high dose WT pork; GE1: low dose GE pork; GE2: high dose GE pork. All data are expressed in mean ± SD from four rats per sex per group.
(DOCX)
