# Supplementary figures and images for: A 90-Day Feeding Study in Rats to Assess the Safety of Genetically Engineered Pork
Source: PLoS One. 2016 Nov 3;11(11):e0165843. doi: 10.1371/journal.pone.0165843 (PMC5094721; doi:10.1371/journal.pone.0165843)

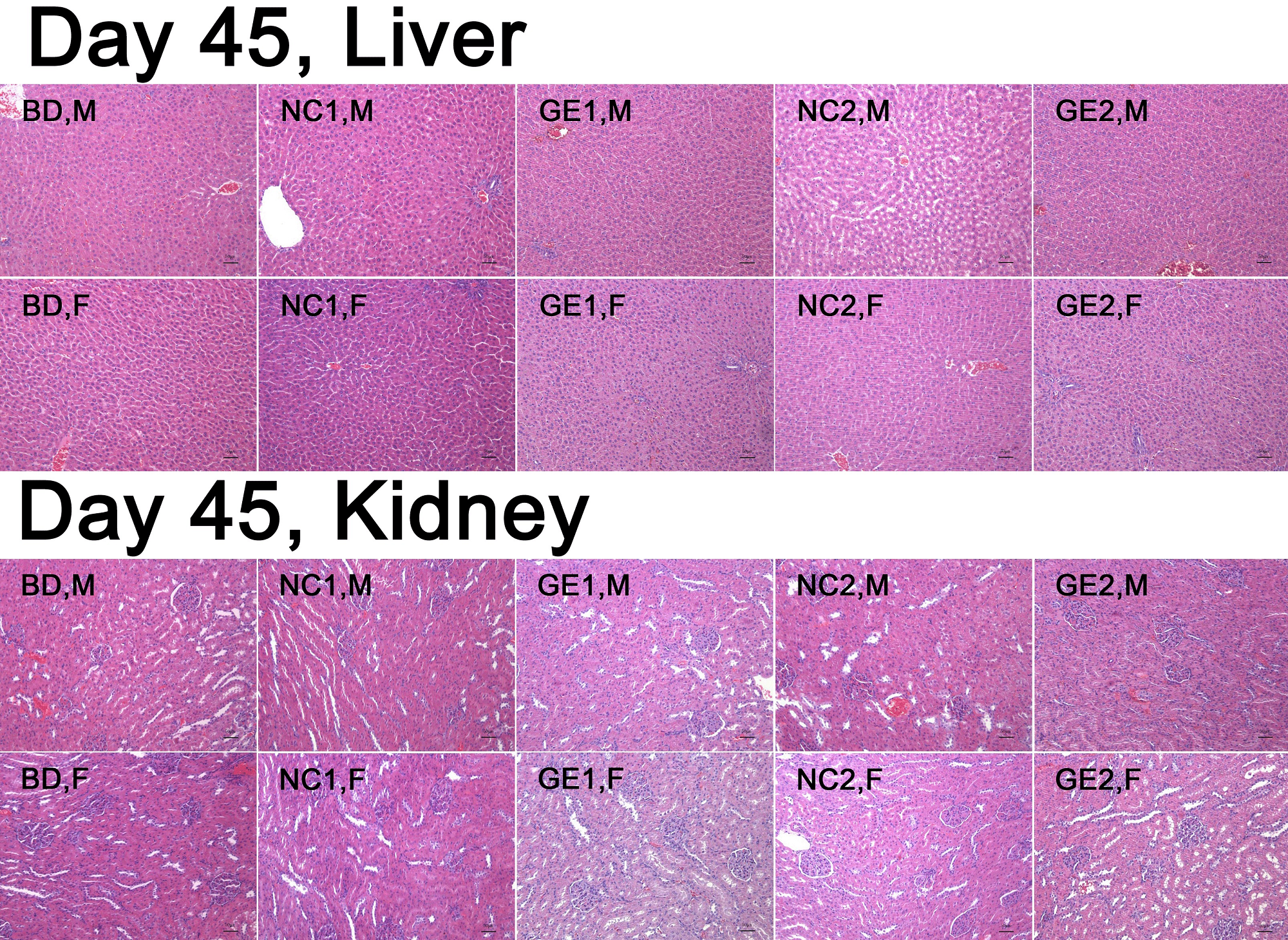

Supplement: S1 Fig — BD: basic diet; NC1: low-dose WT pork; NC2: high dose WT pork; GE1: low dose GE pork; GE2: high dose GE pork. M: male; F: female. (TIF) [file pone.0165843.s001.tif]

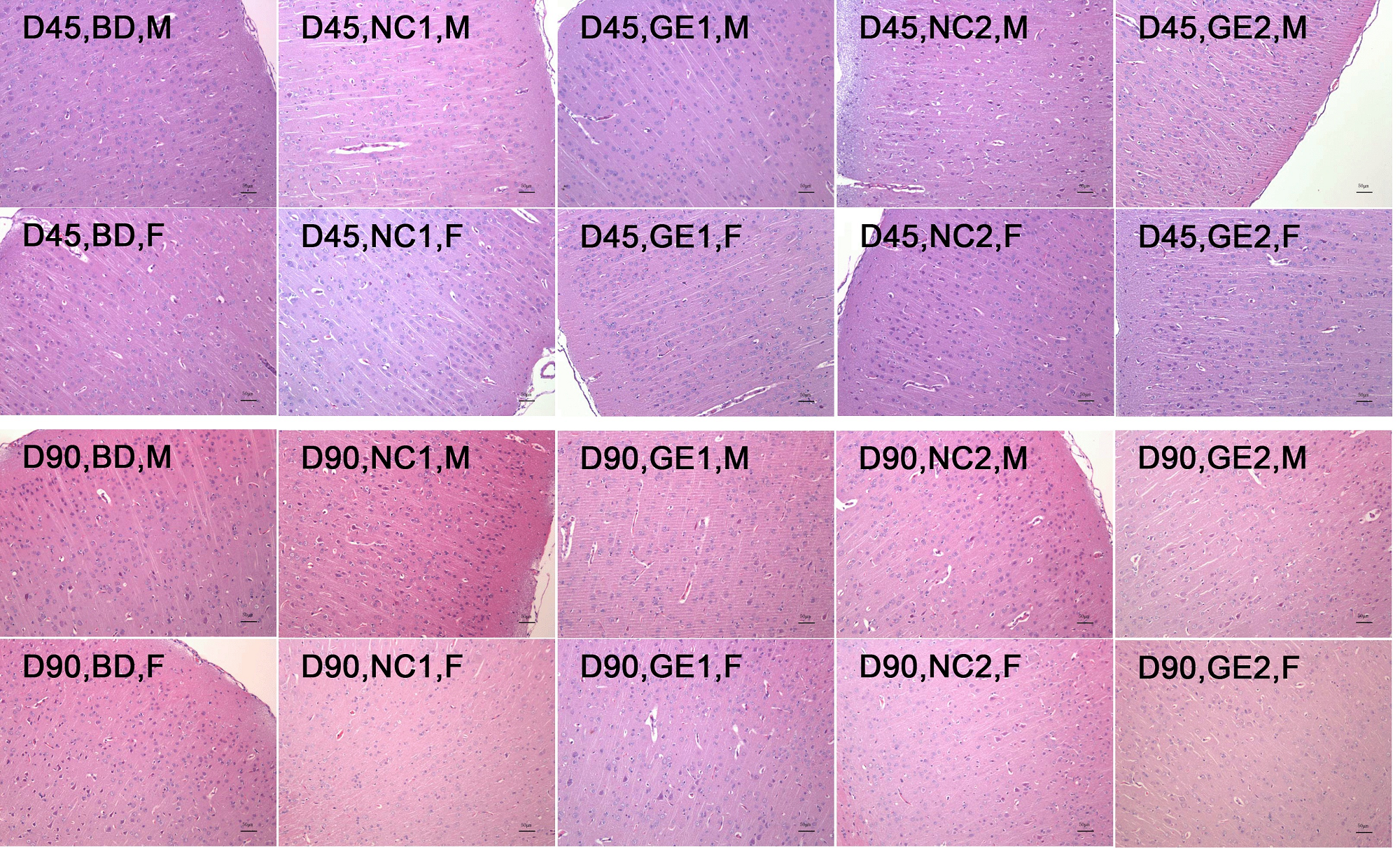

Supplement: S2 Fig — BD: basic diet; NC1: low-dose WT pork; NC2: high dose WT pork; GE1: low dose GE pork; GE2: high dose GE pork. M: male; F: female. (TIF) [file pone.0165843.s002.tif]

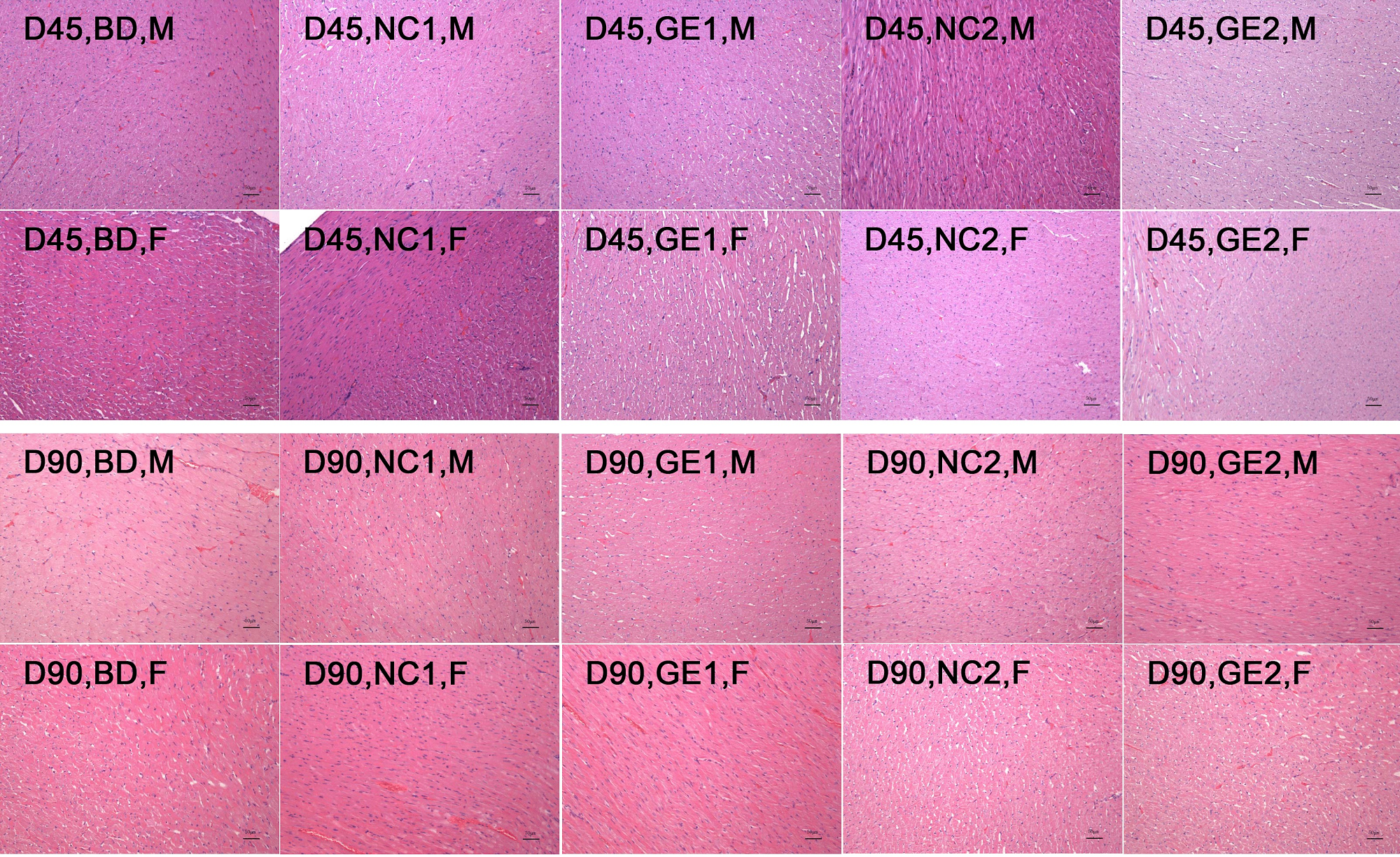

Supplement: S3 Fig — BD: basic diet; NC1: low-dose WT pork; NC2: high dose WT pork; GE1: low dose GE pork; GE2: high dose GE pork. M: male; F: female. (TIF) [file pone.0165843.s003.tif]

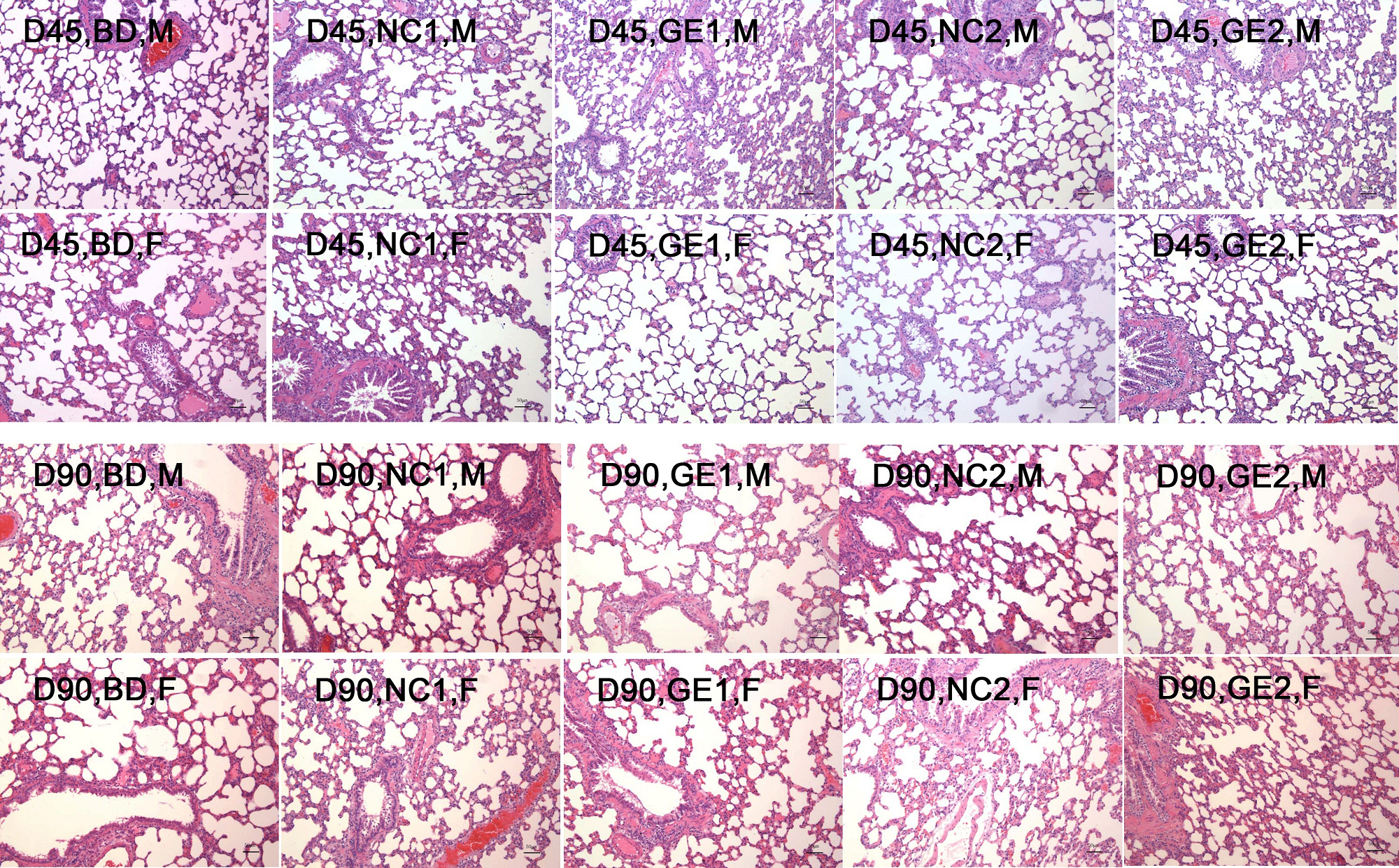

Supplement: S4 Fig — BD: basic diet; NC1: low-dose WT pork; NC2: high dose WT pork; GE1: low dose GE pork; GE2: high dose GE pork. M: male; F: female. (TIF) [file pone.0165843.s004.tif]

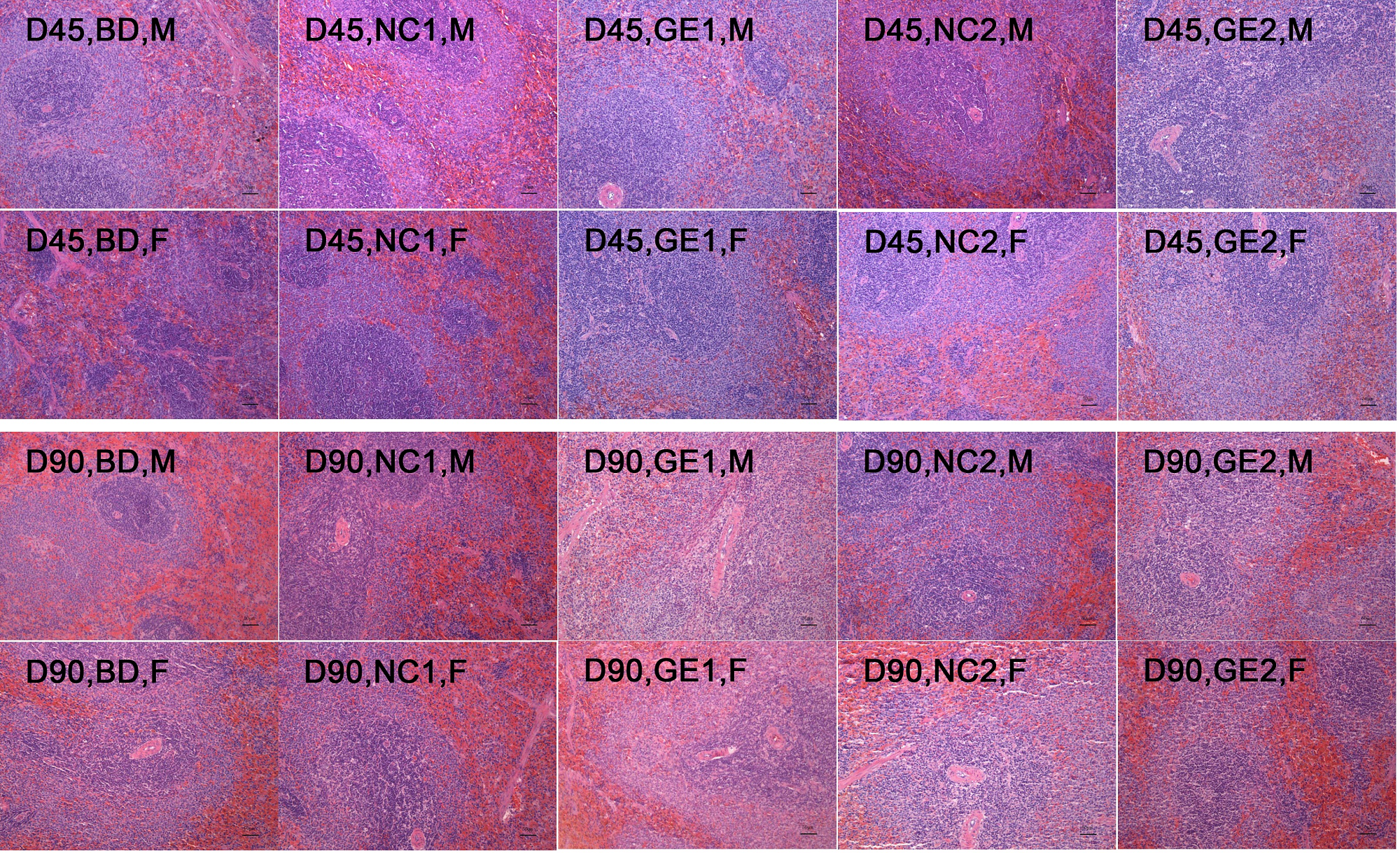

Supplement: S5 Fig — BD: basic diet; NC1: low-dose WT pork; NC2: high dose WT pork; GE1: low dose GE pork; GE2: high dose GE pork. M: male; F: female. (TIF) [file pone.0165843.s005.tif]

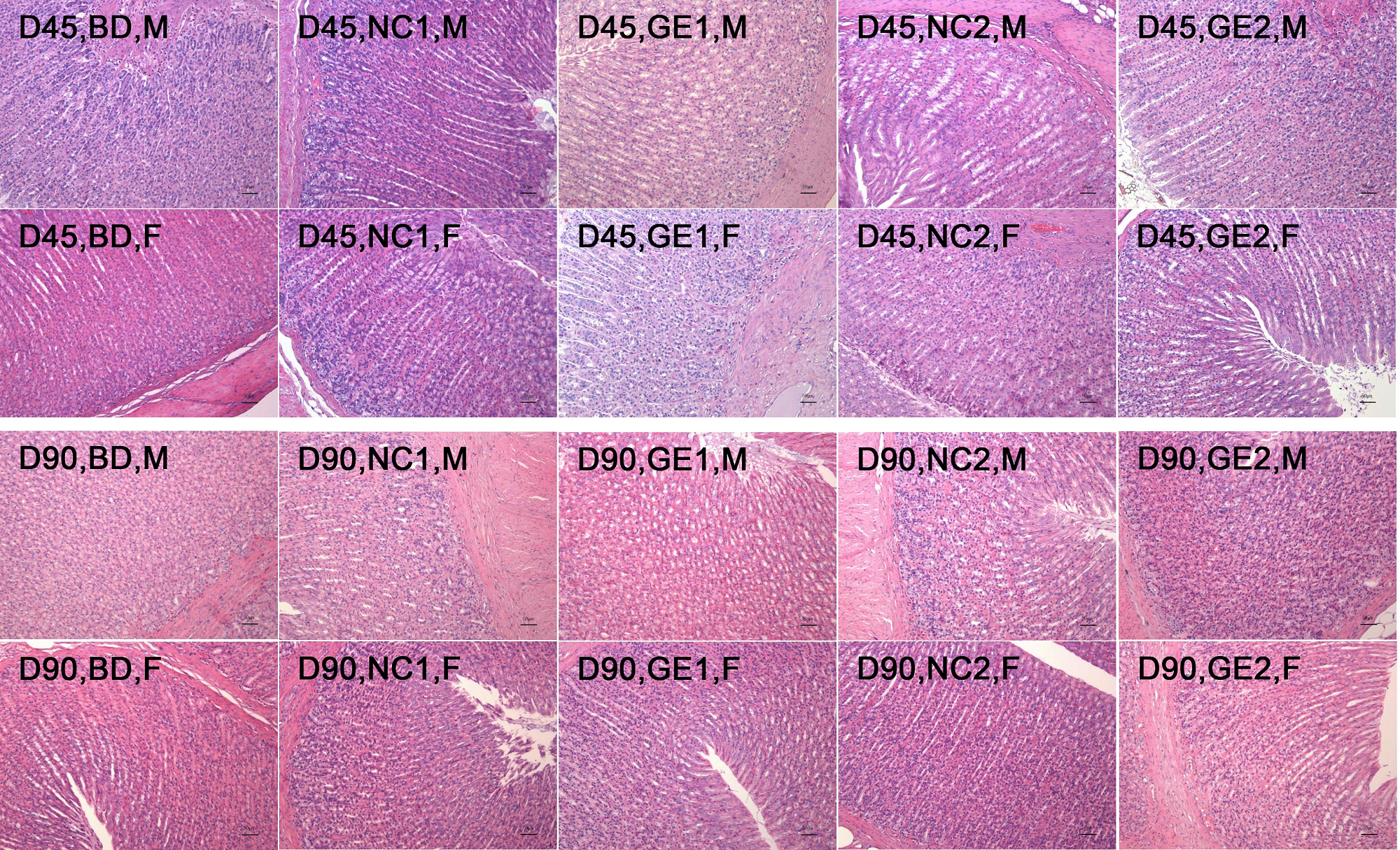

Supplement: S6 Fig — BD: basic diet; NC1: low-dose WT pork; NC2: high dose WT pork; GE1: low dose GE pork; GE2: high dose GE pork. M: male; F: female. (TIF) [file pone.0165843.s006.tif]

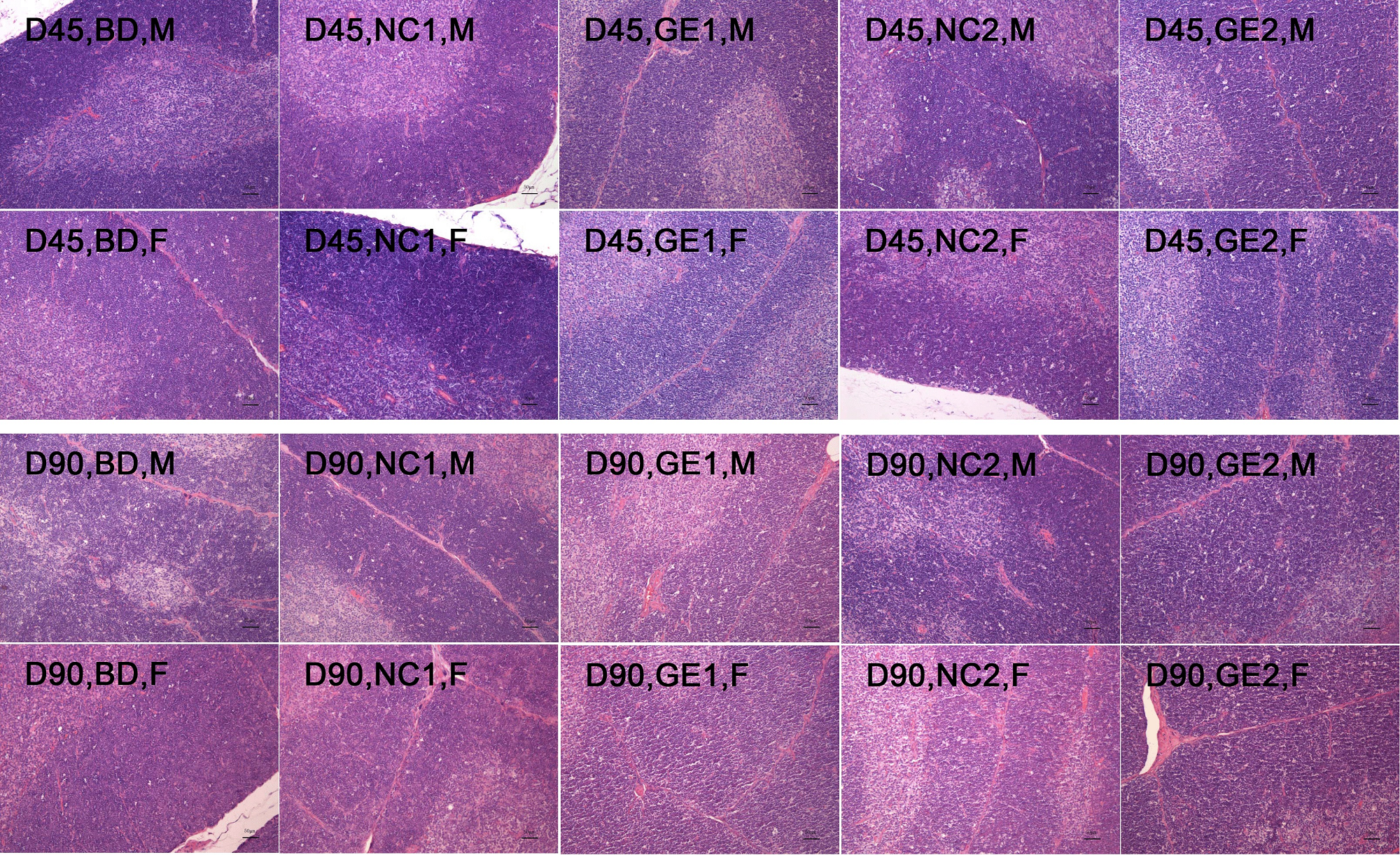

Supplement: S7 Fig — BD: basic diet; NC1: low-dose WT pork; NC2: high dose WT pork; GE1: low dose GE pork; GE2: high dose GE pork. M: male; F: female. (TIF) [file pone.0165843.s007.tif]

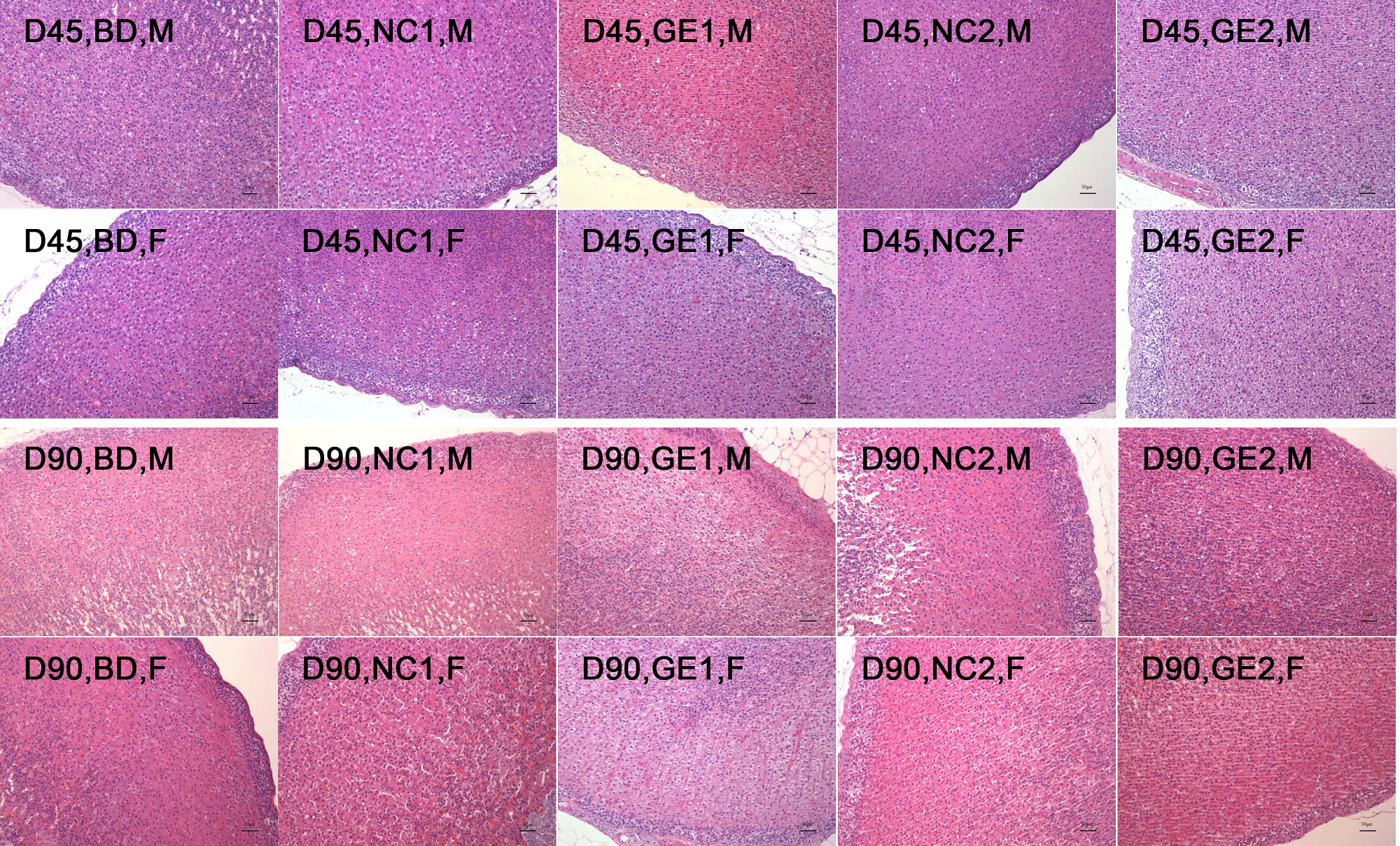

Supplement: S8 Fig — BD: basic diet; NC1: low-dose WT pork; NC2: high dose WT pork; GE1: low dose GE pork; GE2: high dose GE pork. M: male; F: female. (TIF) [file pone.0165843.s008.tif]

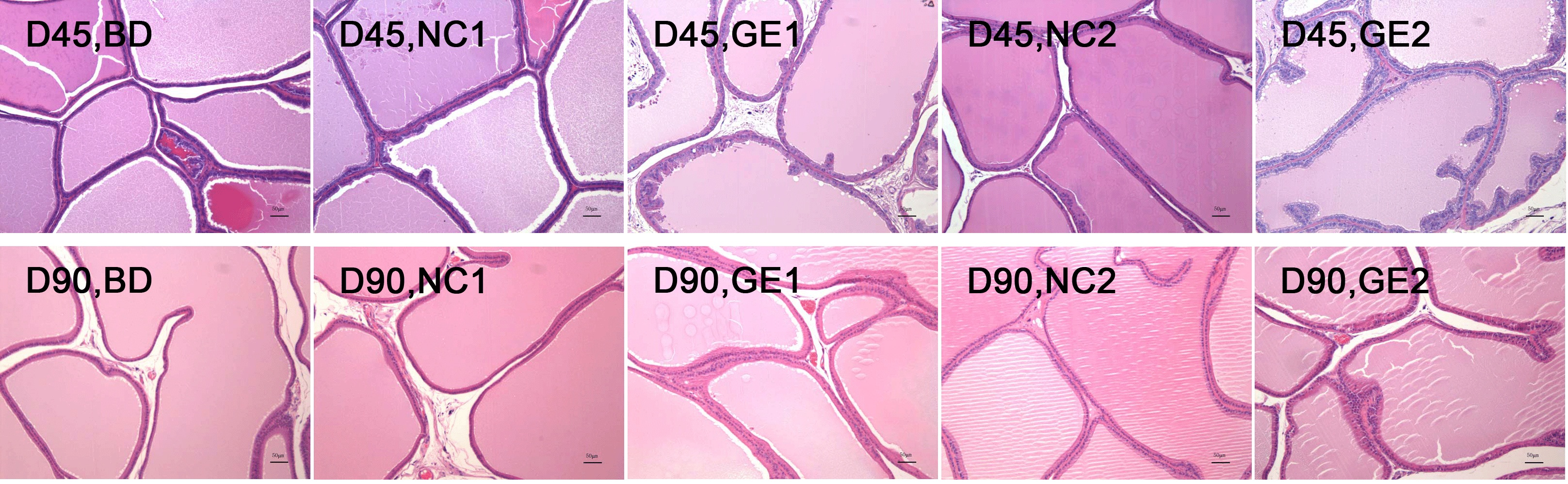

Supplement: S9 Fig — BD: basic diet; NC1: low-dose WT pork; NC2: high dose WT pork; GE1: low dose GE pork; GE2: high dose GE pork. M: male; F: female. (TIF) [file pone.0165843.s009.tif]

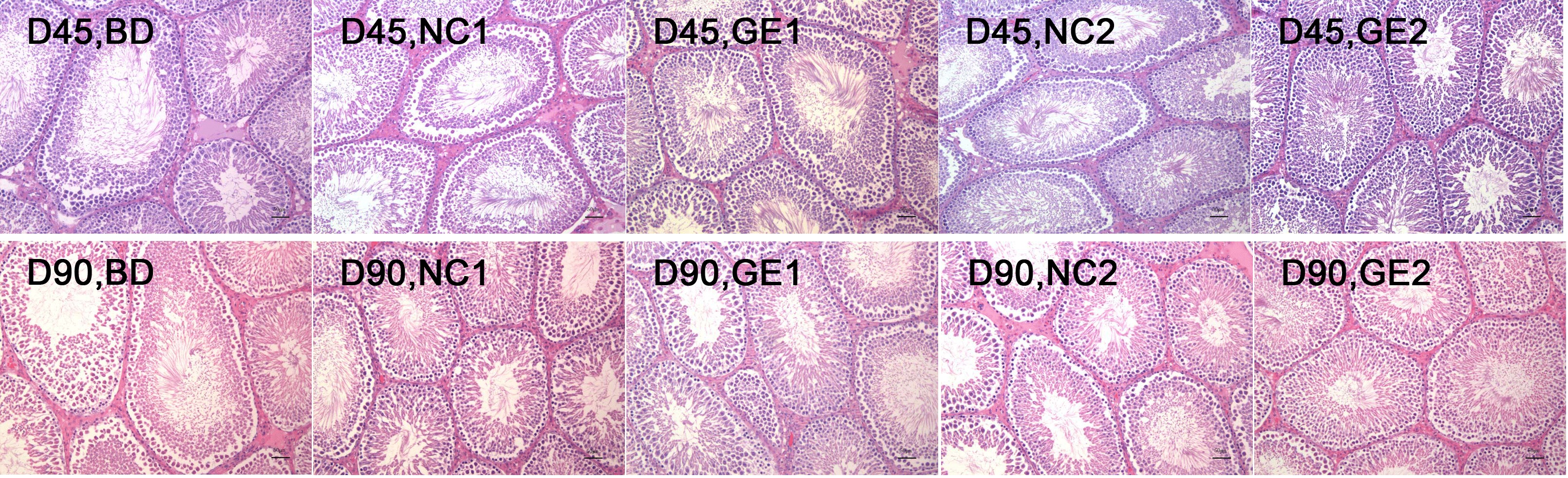

Supplement: S10 Fig — BD: basic diet; NC1: low-dose WT pork; NC2: high dose WT pork; GE1: low dose GE pork; GE2: high dose GE pork. M: male; F: female. (TIF) [file pone.0165843.s010.tif]

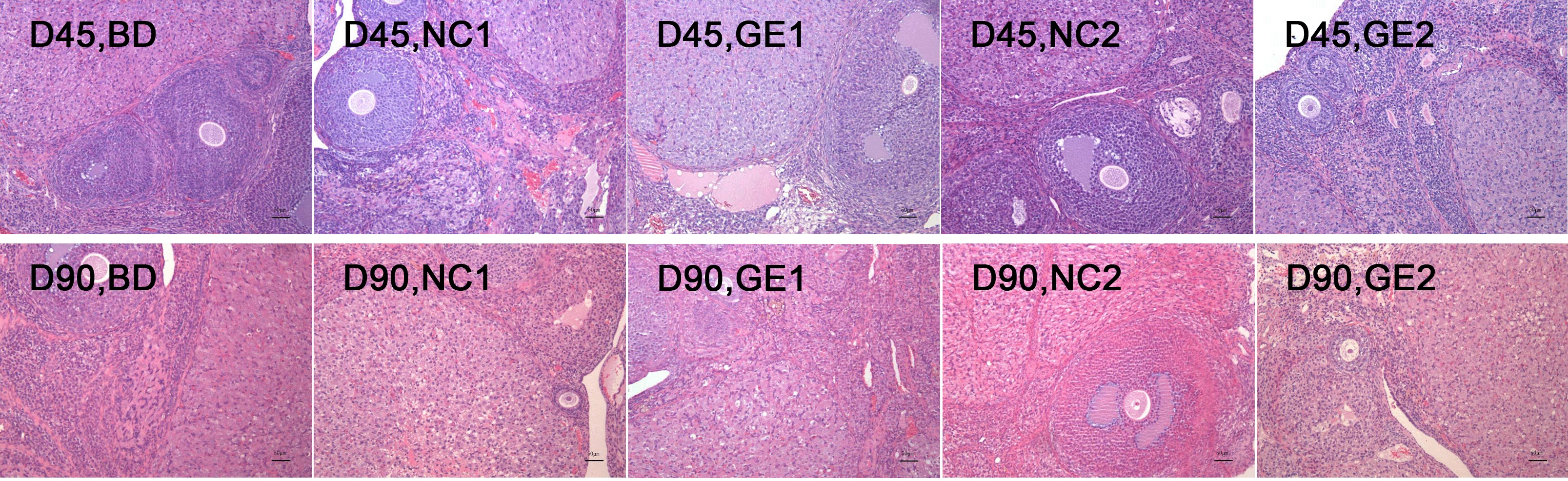

Supplement: S11 Fig — BD: basic diet; NC1: low-dose WT pork; NC2: high dose WT pork; GE1: low dose GE pork; GE2: high dose GE pork. M: male; F: female. (TIF) [file pone.0165843.s011.tif]

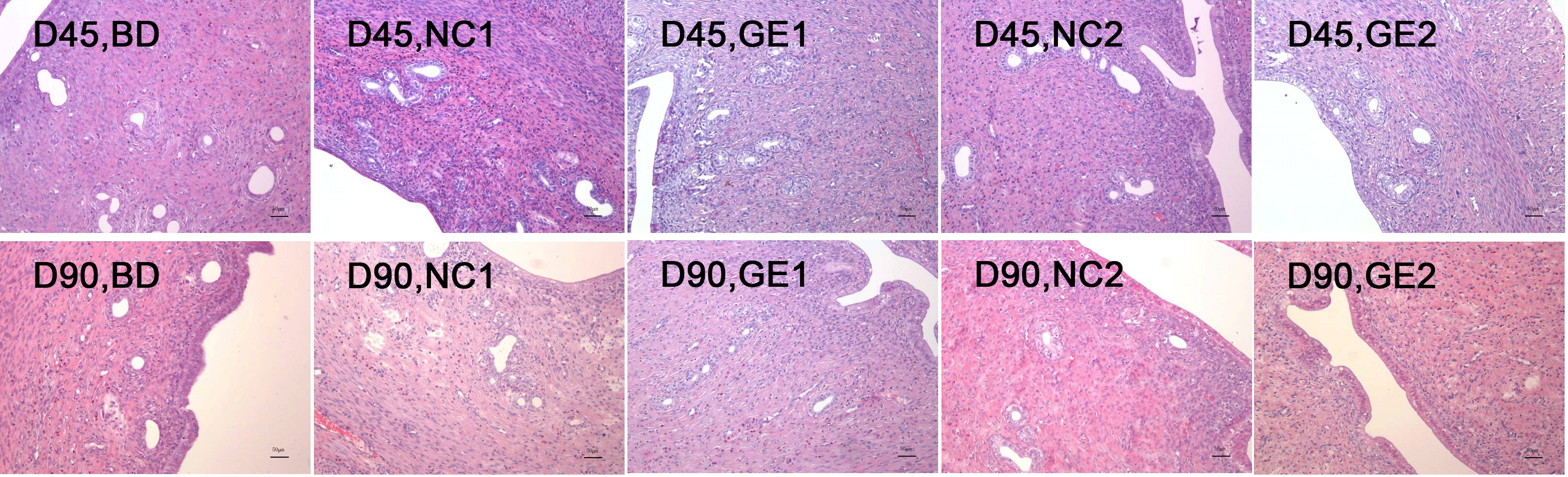

Supplement: S12 Fig — BD: basic diet; NC1: low-dose WT pork; NC2: high dose WT pork; GE1: low dose GE pork; GE2: high dose GE pork. M: male; F: female. (TIF) [file pone.0165843.s012.tif]

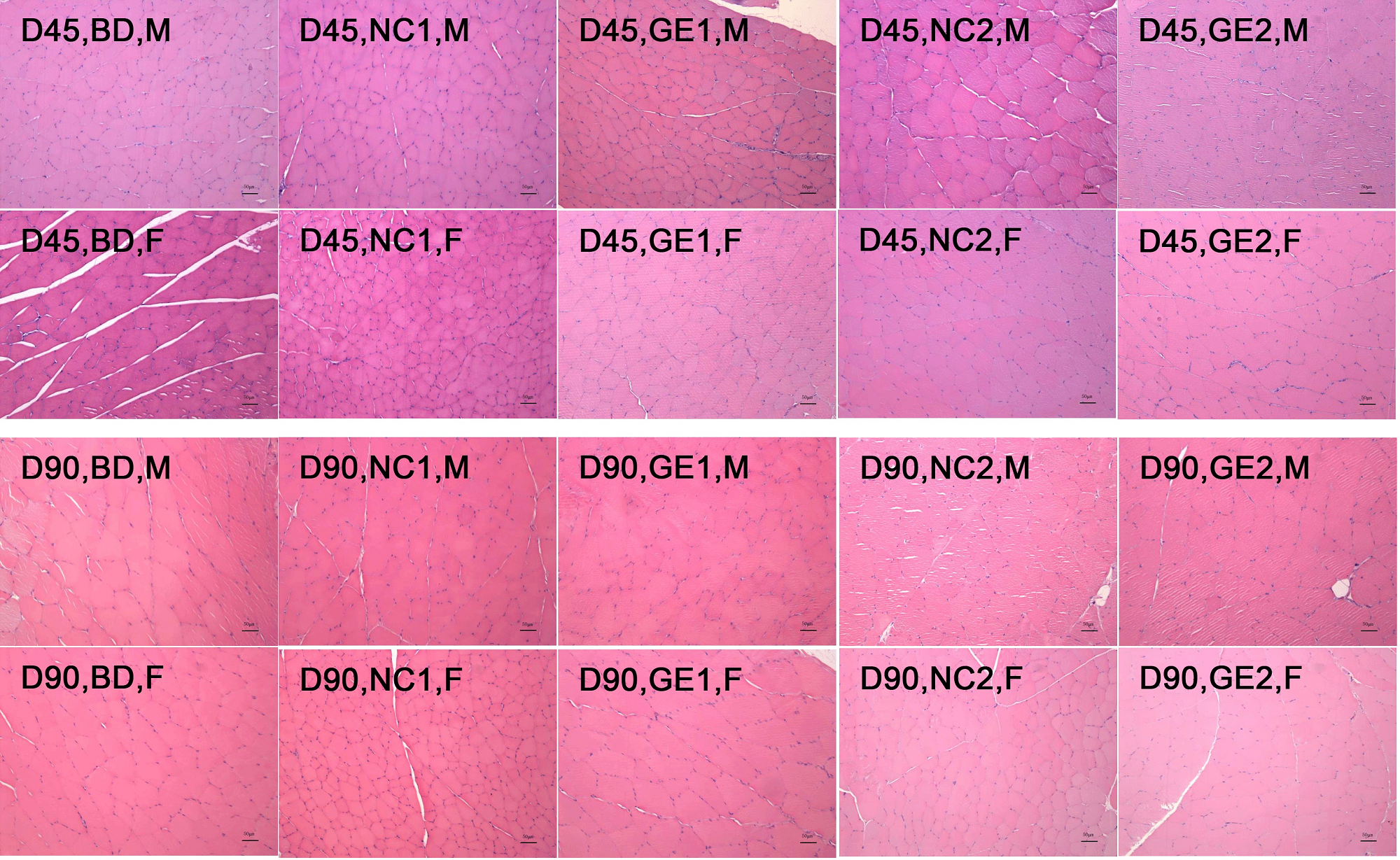

Supplement: S13 Fig — BD: basic diet; NC1: low-dose WT pork; NC2: high dose WT pork; GE1: low dose GE pork; GE2: high dose GE pork. M: male; F: female. (TIF) [file pone.0165843.s013.tif]
