# Supplementary material for: A 90-Day Feeding Study in Rats to Assess the Safety of Genetically Engineered Pork
Source: PLoS One. 2016 Nov 3;11(11):e0165843. doi: 10.1371/journal.pone.0165843 (PMC5094721; doi:10.1371/journal.pone.0165843)
Supplement: S1 Table — GE: genetically engineered; WT: wild type. (DOCX) [file pone.0165843.s014.docx]

**S1 Table, Percentage of amino aicds in pork produced by wild type and GE pigs**

|  | GE-1 | GE-2 | GE-3 | WT-1 | WT-2 | WT-3 |
| --- | --- | --- | --- | --- | --- | --- |
| Asp% | 1.96 | 1.95 | 2.04 | 2.00 | 1.97 | 1.97 |
| Thr% | 1.01 | 1.01 | 1.05 | 1.02 | 1.01 | 1.00 |
| Ser% | 0.84 | 0.83 | 0.85 | 0.85 | 0.83 | 0.83 |
| Glu% | 3.52 | 3.52 | 3.44 | 3.53 | 3.46 | 3.28 |
| Gly% | 0.83 | 0.83 | 0.87 | 0.90 | 0.87 | 1.05 |
| Ala% | 1.17 | 1.16 | 1.19 | 1.19 | 1.17 | 1.22 |
| Cys% | 0.22 | 0.23 | 0.23 | 0.23 | 0.22 | 0.22 |
| Val% | 1.05 | 1.03 | 1.07 | 1.04 | 1.02 | 1.03 |
| Met% | 0.59 | 0.56 | 0.48 | 0.54 | 0.52 | 0.41 |
| Ile% | 1.00 | 0.99 | 1.03 | 1.00 | 1.00 | 0.97 |
| Leu% | 1.74 | 1.72 | 1.80 | 1.76 | 1.72 | 1.70 |
| Tyr% | 0.27 | 0.30 | 0.32 | 0.29 | 0.29 | 0.27 |
| Phe% | 0.88 | 0.87 | 0.91 | 0.90 | 0.87 | 0.89 |
| Lys% | 1.93 | 1.91 | 2.00 | 1.94 | 1.91 | 1.90 |
| His% | 0.98 | 1.01 | 1.07 | 1.00 | 0.98 | 0.97 |
| Arg% | 1.34 | 1.34 | 1.40 | 1.40 | 1.36 | 1.37 |
| Pro% | 0.52 | 0.53 | 0.56 | 0.58 | 0.57 | 0.67 |

GE: genetically engineered; WT: wild type
