# Supplementary material for: A 90-Day Feeding Study in Rats to Assess the Safety of Genetically Engineered Pork
Source: PLoS One. 2016 Nov 3;11(11):e0165843. doi: 10.1371/journal.pone.0165843 (PMC5094721; doi:10.1371/journal.pone.0165843)
Supplement: S2 Table — BD: basic diet; NC1: low-dose WT pork; NC2: high dose WT pork; GE1: low dose GE pork; GE2: high dose GE pork. All data are expressed in mean ± SD from four rats per sex per group. (DOCX) [file pone.0165843.s015.docx]

**S2 Table, Test results (mean ± SD) of serum lipid panel at day45**

|  | Test results at day 45 | | | | |
| --- | --- | --- | --- | --- | --- |
|  | BD | NC1 | NC2 | GE1 | GE2 |
| Male rats | | | | | |
| TG | 0.67±0.23 | 0.48±0.11 | 0.58±0.11 | 0.53±0.02 | 0.77±0.15 |
| CHO | 1.94±0.50 | 1.55±0.29 | 1.96±0.27 | 1.48±0.01 | 1.87±0.13 |
| HDL-C | 1.30±0.31 | 0.93±0.29 | 1.43±0.24 | 0.95±0.04 | 1.25±0.11 |
| LDL-C | 0.17±0.05 | 0.15±0.06 | 0.19±0.03 | 0.16±0.03 | 0.16±0.02 |
| Female rats | | | | | |
| TG | 0.45±0.03 | 0.41±0.01 | 0.49±0.03 | 0.49±0.05 | 0.44±0.05 |
| CHO | 2.01±0.44 | 1.92±0.03 | 2.41±0.48 | 2.16±0.23 | 2.17±0.11 |
| HDL-C | 1.41±0.33 | 1.32±0.06 | 1.84±0.35 | 1.65±0.29 | 1.41±0.28 |
| LDL-C | 0.19±0.04 | 0.19±0.02 | 0.19±0.03 | 0.20±0.01 | 0.18±0.02 |

BD: basic diet; NC1: low-dose WT pork; NC2: high dose WT pork; GE1: low dose GE pork; GE2: high dose GE pork. All data are expressed in mean ± SD from four rats per sex per group.
