# Supplementary material for: A 90-Day Feeding Study in Rats to Assess the Safety of Genetically Engineered Pork
Source: PLoS One. 2016 Nov 3;11(11):e0165843. doi: 10.1371/journal.pone.0165843 (PMC5094721; doi:10.1371/journal.pone.0165843)
Supplement: S3 Table — BD: basic diet; NC1: low-dose WT pork; NC2: high dose WT pork; GE1: low dose GE pork; GE2: high dose GE pork. All data are expressed in mean ± SD from four rats per sex per group. (DOCX) [file pone.0165843.s016.docx]

**S3 Table, Test results (mean ± SD) of serum electrolytes at day 45**

|  | Test results at day 45 | | | | |
| --- | --- | --- | --- | --- | --- |
|  | BD | NC1 | NC2 | GE1 | GE2 |
| Male rats | | | | | |
| K | 4.35±0.34 | 4.28±0.15 | 4.50±0.08 | 4.38±0.26 | 4.70±0.47 |
| Na | 141.25±0.96 | 142.0±0.82 | 142.5±0.58 | 141.0±0.58 | 142.0±0.0 |
| Ca | 2.49±0.02 | 2.46±0.04 | 2.49±0.03 | 2.47±0.05 | 2.50±0.05 |
| Cl | 103.25±1.50 | 103.67±0.58 | 104.25±0.96 | 102.67±0.58 | 104.0±0.82 |
| Female rats | | | | | |
| K | 4.40±0.22 | 4.28±0.33 | 4.30±0.17 | 4.23±0.21 | 4.63±0.15 |
| Na | 140.67±0.58 | 140.67±0.58 | 141.0±0.0 | 140.67±1.53 | 140.33±0.58 |
| Ca | 2.48±0.04 | 2.44±0.06 | 2.47±0.08 | 2.49±0.09 | 2.44±0.04 |
| Cl | 105.0±1.41 | 105.25±1.50 | 105.25±1.71 | 104.50±1.73 | 104.33±1.53 |

BD: basic diet; NC1: low-dose WT pork; NC2: high dose WT pork; GE1: low dose GE pork; GE2: high dose GE pork. All data are expressed in mean ± SD from four rats per sex per group.
