# Supplementary material for: A 90-Day Feeding Study in Rats to Assess the Safety of Genetically Engineered Pork
Source: PLoS One. 2016 Nov 3;11(11):e0165843. doi: 10.1371/journal.pone.0165843 (PMC5094721; doi:10.1371/journal.pone.0165843)
Supplement: S4 Table — BD: basic diet; NC1: low-dose WT pork; NC2: high dose WT pork; GE1: low dose GE pork; GE2: high dose GE pork. All data are expressed in mean ± SD from four rats per sex per group. (DOCX) [file pone.0165843.s017.docx]

**S4 Table, Test results (mean ± SD) of blood parameters related to liver function serum electrolytes at day 45**

|  | Test results at day 45 | | | | |
| --- | --- | --- | --- | --- | --- |
|  | BD | NC1 | NC2 | GE1 | GE2 |
| Male rats | | | | | |
| ALT | 34.00±4.00 | 28.67±3.79 | 33.33±3.79 | 30.33±3.51 | 32.67±2.08 |
| AST | 81.25±7.23 | 74.5±7.14 | 82.67±8.14 | 75.67±3.21 | 78.67±3.06 |
| ALP | 118.67±5.51 | 102.33±11.50 | 118.33±8.14 | 105.67±13.61 | 110.0±9.17 |
| AST/ALT | 2.02±0.35 | 2.81±0.11 | 2.56±0.43 | 2.67±0.25 | 2.55±0.08 |
| TP | 55.75±3.07 | 52.92±1.87 | 53.86±0.89 | 53.05±1.26 | 53.84±1.65 |
| ALB | 30.41±1.00 | 29.34±0.22 | 29.34±0.89 | 28.42±1.22 | 28.92±0.46 |
| GLOB | 26.42±0.50 | 25.31±1.07 | 24.93±0.88 | 25.45±0.86 | 26.14±0.26 |
| A/G | 1.10±0.07 | 1.17±0.04 | 1.24±0.08 | 1.13±0.02 | 1.13±0.08 |
| Female rats | | | | | |
| ALT | 29.0±1.73 | 29.75±2.75 | 26.25±2.22 | 30.33±6.11 | 26.0±2.65 |
| AST | 129.0±21.0 | 118.67±11.06 | 92.33±18.93 | 86.0±17.58 | 101.33±13.65 |
| ALP | 58.67±20.21 | 60.5±4.04 | 69.33±18.04 | 71.67±22.81 | 74.33±9.81 |
| AST/ALT | 3.56±0.48 | 3.83±0.12 | 3.52±0.87 | 3.42±0.20 | 3.45±0.19 |
| TP | 59.70±2.33 | 56.57±2.68 | 58.29±4.05 | 59.03±5.98 | 56.79±2.87 |
| ALB | 33.68±2.67 | 31.27±1.43 | 35.72±4.46 | 35.86±3.64 | 30.49±3.36 |
| GLOB | 26.53±1.25 | 24.45±1.87 | 25.07±0.76 | 24.92±2.72 | 25.25±1.68 |
| A/G | 1.25±0.11 | 1.28±0.07 | 1.45±0.12 | 1.44±0.08 | 1.21±0.16 |

BD: basic diet; NC1: low-dose WT pork; NC2: high dose WT pork; GE1: low dose GE pork; GE2: high dose GE pork. All data are expressed in mean ± SD from four rats per sex per group.
