# Supplementary material for: A 90-Day Feeding Study in Rats to Assess the Safety of Genetically Engineered Pork
Source: PLoS One. 2016 Nov 3;11(11):e0165843. doi: 10.1371/journal.pone.0165843 (PMC5094721; doi:10.1371/journal.pone.0165843)
Supplement: S5 Table — BD: basic diet; NC1: low-dose WT pork; NC2: high dose WT pork; GE1: low dose GE pork; GE2: high dose GE pork. All data are expressed in mean ± SD from four rats per sex per group. (DOCX) [file pone.0165843.s018.docx]

**S5 Table, Results (mean ± SD) of liver weight and liver weight coefficient (liver weight /body weight ratio) at days 45**

|  | Test results at day 45 | | | | |
| --- | --- | --- | --- | --- | --- |
|  | BD | NC1 | NC2 | GE1 | GE2 |
| Male rats | | | | | |
| Liver | 11.61±1.29 | 10.36±0.36 | 12.55±0.53 | 11.53±1.47 | 13.16±0.71 |
| Body | 420.28±35.55 | 407.50±26.48 | 438.23±26.55 | 457.53±14.48 | 420.28±35.55 |
| Coefficient | 0.028±0.001 | 0.026±0.002 | 0.029±0.001 | 0.026±0.002 | 0.029±0.001 |
| Female rats | | | | | |
| Liver | 7.68±0.61 | 8.29±0.78 | 7.88±0.73 | 8.57±0.46 | 7.64±1.05 |
| Body | 277.33±11.83 | 185.55±32.48 | 288.08±29.74 | 305.00±21.97 | 266.10±14.65 |
| Coefficient | 0.028±0.001 | 0.029±0.001 | 0.027±0.001 | 0.028±0.001 | 0.029±0.002 |

BD: basic diet; NC1: low-dose WT pork; NC2: high dose WT pork; GE1: low dose GE pork; GE2: high dose GE pork. All data are expressed in mean ± SD from four rats per sex per group.
