# Supplementary material for: A 90-Day Feeding Study in Rats to Assess the Safety of Genetically Engineered Pork
Source: PLoS One. 2016 Nov 3;11(11):e0165843. doi: 10.1371/journal.pone.0165843 (PMC5094721; doi:10.1371/journal.pone.0165843)
Supplement: S6 Table — BD: basic diet; NC1: low-dose WT pork; NC2: high dose WT pork; GE1: low dose GE pork; GE2: high dose GE pork. All data are expressed in mean ± SD from four rats per sex per group. (DOCX) [file pone.0165843.s019.docx]

**S6 Table, Test results (mean ± SD) of blood parameters related to renal function at days 45.**

|  | Test results at day 45 | | | | |
| --- | --- | --- | --- | --- | --- |
|  | BD | NC1 | NC2 | GE1 | GE2 |
| Male rats | | | | | |
| BUN | 5.97±0.76 | 5.50±0.38 | 5.29±0.66 | 6.40±0.68 | 6.04±0.54 |
| CREA | 18.12±1.91 | 18.01±1.73 | 18.51±2.01 | 17.78±3.04 | 18.61±1.43 |
| GLU | 6.69±0.99 | 5.56±0.68 | 647±0.56 | 6.40±0.78 | 6.73±0.58 |
| Female rats | | | | | |
| BUN | 5.99±0.46 | 5.42±0.05 | 5.42±0.68 | 5.28±0.29 | 5.35±0.68 |
| CREA | 23.66±2.31 | 23.78±2.22 | 28.24±4.19 | 23.09±2.91 | 24.19±1.55 |
| GLU | 6.40±0.79 | 6.19±0.30 | 6.98±0.28 | 6.09±0.51 | 6.85±0.36 |

BD: basic diet; NC1: low-dose WT pork; NC2: high dose WT pork; GE1: low dose GE pork; GE2: high dose GE pork. All data are expressed in mean ± SD from four rats per sex per group.
