# Supplementary material for: A 90-Day Feeding Study in Rats to Assess the Safety of Genetically Engineered Pork
Source: PLoS One. 2016 Nov 3;11(11):e0165843. doi: 10.1371/journal.pone.0165843 (PMC5094721; doi:10.1371/journal.pone.0165843)
Supplement: S7 Table — BD: basic diet; NC1: low-dose WT pork; NC2: high dose WT pork; GE1: low dose GE pork; GE2: high dose GE pork. All data are expressed in mean ± SD from four rats per sex per group. (DOCX) [file pone.0165843.s020.docx]

**S7 Table, Results (mean ± SD) of kidney weight and kidney weight coefficient (liver weight /body weight ratio) at days 45**

|  | Test results at day 45 | | | | |
| --- | --- | --- | --- | --- | --- |
|  | BD | NC1 | NC2 | GE1 | GE2 |
| Male rats | | | | | |
| Kidney | 2.79±0.24 | 3.05±0.11 | 3.36±0.13 | 2.77±0.24 | 3.43±0.29 |
| Body | 420.28±35.55 | 407.50±26.48 | 438.23±26.55 | 457.53±14.48 | 420.28±35.55 |
| Coefficient | 0.0066±0.0001 | 0.0073±0.0006 | 0.0076±0.0002 | 0.0063±0.0004 | 0.0075±0.0006 |
| Female rats | | | | | |
| Kidney | 1.97±0.16 | 2.07±0.22 | 1.97±0.15 | 2.04±0.09 | 2.01±0.26 |
| Body | 277.33±11.83 | 185.55±32.48 | 288.08±29.74 | 305.00±21.97 | 266.10±14.65 |
| Coefficient | 0.0071±0.0003 | 0.0073±0.0009 | 0.0069±0.0005 | 0.0067±0.0007 | 0.0075±0.0006 |

BD: basic diet; NC1: low-dose WT pork; NC2: high dose WT pork; GE1: low dose GE pork; GE2: high dose GE pork. All data are expressed in mean ± SD from four rats per sex per group.
